# Supplementary material for: Supersulphides provide airway protection in viral and chronic lung diseases
Source: Nat Commun. 2023 Jul 25;14:4476. doi: 10.1038/s41467-023-40182-4 (PMC10368687; doi:10.1038/s41467-023-40182-4)
Supplement: Supplementary file 1 — Supplementary figure [file 41467_2023_40182_MOESM1_ESM.pdf]

Supplementary Figure 1

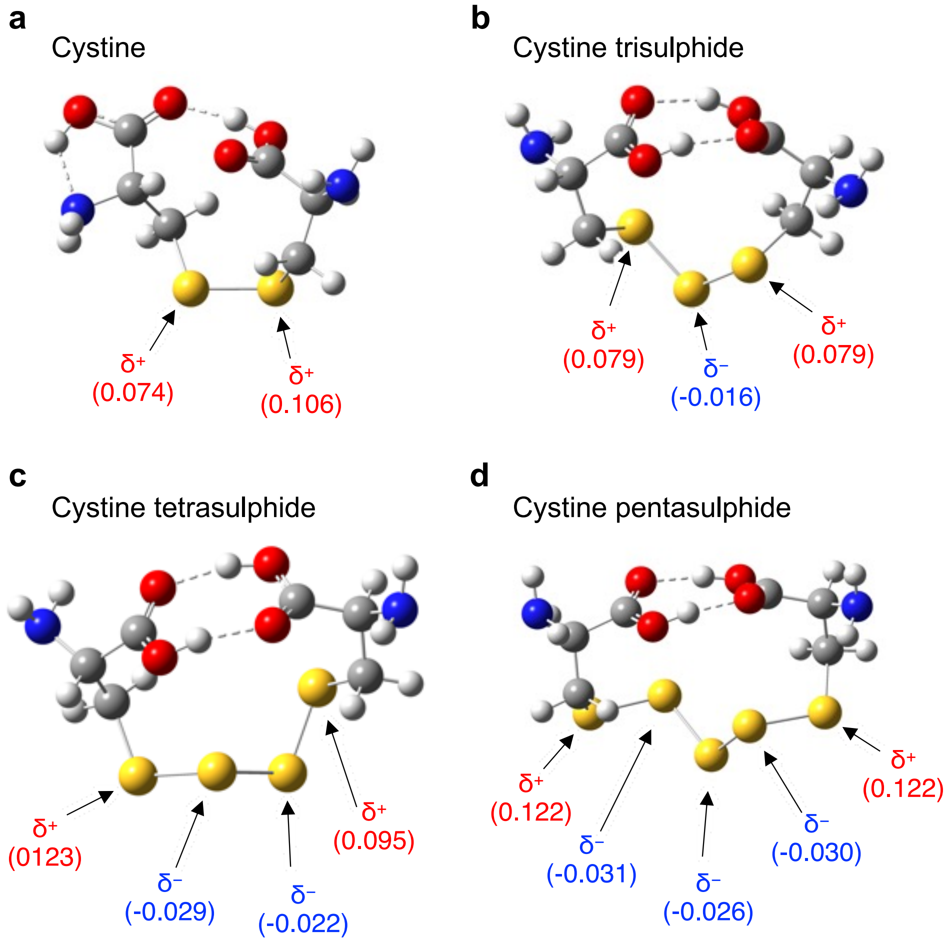

Supplementary Fig. 1 Physicochemical and biochemical properties of supersulphides. We currently define "supersulphides" as hydropersulphide (RSSH) species and polymeric sulphurs with sulphur catenation (RSS_nR, $n > 1$, R = hydrogen or alkyl, or cyclized sulphurs), which are recognized as universal bioactive metabolites formed physiologically in all organisms[1-4]. Supersulphides include all biologically relevant sulphur compounds and allotropes, which cannot be fully covered by other terms such as reactive sulphur species (RSS), sulphane sulphurs, and persulphides/polysulphides. Regarding the physicochemical property of supersulphides, for example, the charge density was calculated for sulphur catenation residues of supersulphides, and was illustrated by the representative optimized structures of neutral forms of cystine (**a**), cystine trisulphide (**b**), cystine tetrasulphide (**c**), and cystine pentasulphide (**d**) obtained by using B3LYP/6-311 G(2d,p). Conformations at the lowest free energy levels are shown. Within the sulphur catenation chain, sulphur atoms at

the center tend to become more nucleophilic than their flanking sulphur atoms, and those at the end of the chains are electrophilic (**b-d**). In contrast, a simple cysteine disulphide (cystine) is generally electrophilic (**a**). Cystine has a stable structure when one of the hydroxyl groups forms a hydrogen bond with the nitrogen atom in the same cysteine, and the remaining carbonyl group is hydrogen bonded to the hydroxyl group of another cysteine (**a**). Trisulphide, tetrasulphide, and pentasulphide, however, are much more firmly stabilized than simple cystine via steady formation of cyclic dimers, when the two carboxyl groups form hydrogen bonds with each other, in the same fashion as dimers of acetic acid and formic acid (**b-d**). Two intramolecular hydrogen bonds between the hydroxyl hydrogen and the carbonyl oxygen stabilize the structures of the trisulphides, tetrasulphides, and pentasulphides.

The most important biochemical properties of supersulphides are antioxidant and anti-inflammatory properties[1,5]. We previously reported a strong antioxidant effect of persulphides and polysulphides and clarified the strong hydrogen peroxide (H_2O_2)-scavenging activity of GSSH, compared with parental glutathione (GSH) and H_2S, which all failed to eliminate H_2O_2 under the same reaction conditions[1]. Nonetheless, it is also known that H_2O_2 could sulphenylate cysteine residues, which are thereby persulphidated by H_2S[6]; besides persulphidated protein could react with H_2S faster than with glutathione by two orders of magnitude[7]. Li et al. also reported that GSSH was 50 times more reactive than H_2S against H_2O_2 at physiological pH[8]. Everett and Wardman reported that persulphides efficiently scavenged free radical species[9]. Persulphides are stronger acids than are thiols, so at physiological pH (at which many thiols occur predominantly in the protonated form), a significant proportion of hydropersulphide species exist as deprotonated persulphide anions (RSS^-)[10]. This idea is supported by a recent report by Li et al., who stated that the pK_a of GSSH was 6.9, two orders of magnitude smaller than that of GSH (pK_a = 8.9)[8]. RSS^- thus formed reacted readily with radical species to form persulphide radicals (RSS•)[11]. RSS• are considerably less reactive compared with their thiyl radical counterparts and hence are probably less toxic if produced in cells. Supersulphides can also efficiently react with various electrophiles[1,12-15]. These findings demonstrate the redox-active potency of hydropersulphides and polysulphides as strong reductants and nucleophiles: they manifest greater reactivity than thiols and can scavenge oxidants and cellular electrophiles, thereby serving as important antioxidant molecules[1,4,12]. Calculations of the charge density of supersulphides (e.g. cysteine polysulphides shown here) indicate that the sulphur in the middle

sulphur residue in the chain structure tends to become more nucleophilic than the sulphur at the end of a sulphur chain, which is rather electrophilic; a simple cysteine disulphide is generally electrophilic. Our present study first verifies a biologically relevant nucleophilic and antioxidant capacity of GSSSG, and provide a clear demonstration that supersulphides, even present as apparently oxidized forms, still have significant nucleophilic and antioxidant properties.

Supplementary Figure 2

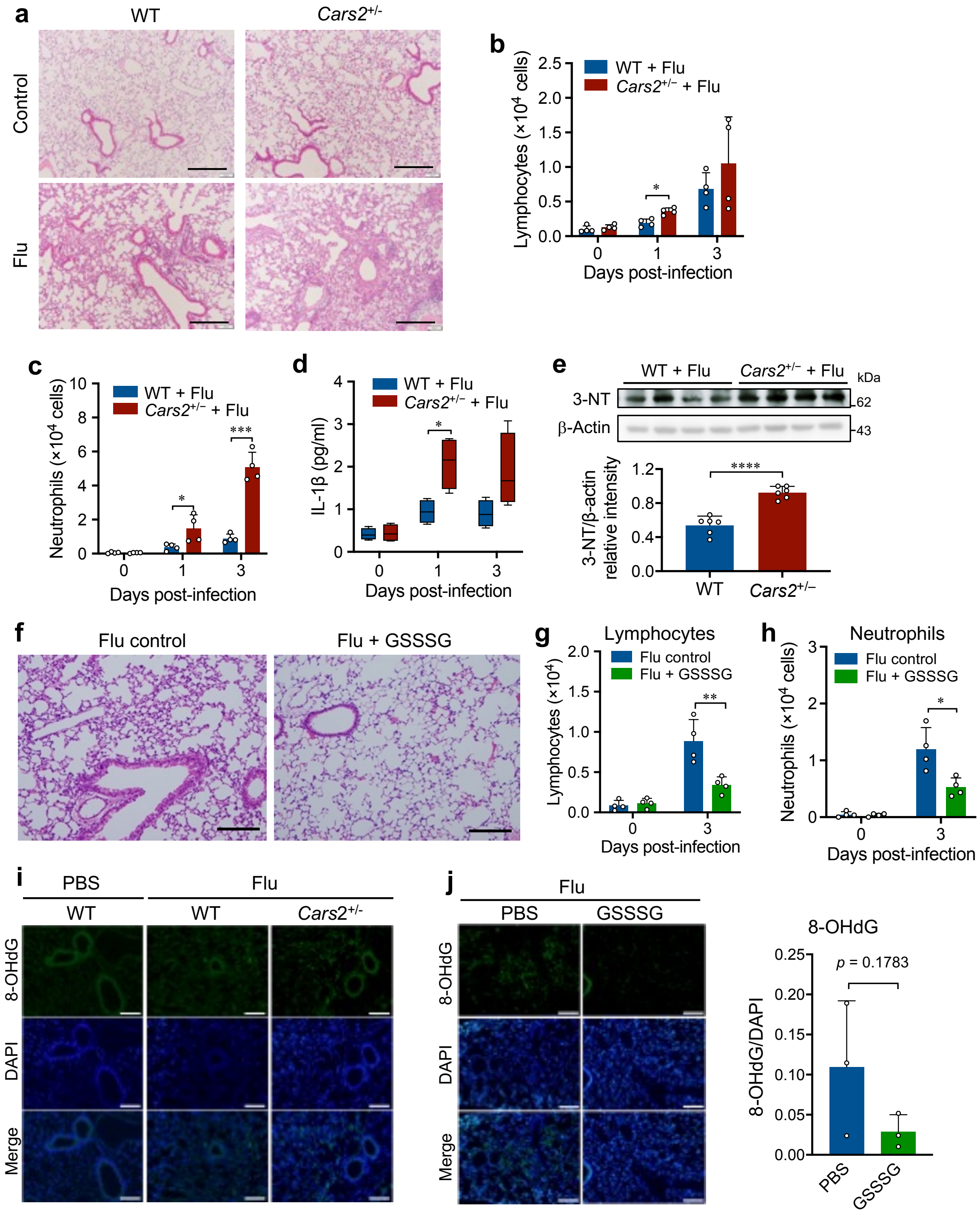

Supplementary Fig. 2 Enhanced influenza virus pathogenesis (inflammation and oxidative/nitrative stress) in lungs of *Cars2*$^{+/–}$ mice.

a, Representative HE-stained images showing pathological changes in lungs of WT and *Cars2*$^{+/–}$ mice at day 3 after influenza virus infection. Scale bars, 100 μm. **b** and **c**,

Numbers of lymphocytes (**b**) and neutrophils (**c**) in BALF from Flu-infected WT and *Cars2*$^{+/-}$ mice. Each dot represents data from an individual mouse (n = 4 per group). P = 0.021 in **b**. P = 0.040 and 0.0004 in **c**. **d**, IL-1β concentrations in BALF from Flu-infected WT and *Cars2*$^{+/-}$ mice (n = 4 per group). P = 0.031. **e**, Production level of 3-nitrotyrosine (3-NT) in lungs from Flu-infected WT and *Cars2*$^{+/-}$ mice as evaluated by Western blotting (upper panel); quantitative results appear in the lower panel (n = 6 per group). β-Actin was used as the loading control. $P < 0.0001$. **f**, Representative HE-stained images showing pathological changes in lungs of WT mice at day 8 after influenza virus infection, with PBS or GSSSG administration. Scale bars, 100 µm. **g** and **h**, Numbers of lymphocytes (**g**) and neutrophils (**h**) in BALF from Flu-infected WT mice treated with PBS or GSSSG (n = 4 per group). P = 0.0091 in **g**. P = 0.017 in **h**. **i**, Representative images of 8-hydroxy-2′-deoxyguanosine (8-OHdG) immunostaining in lungs in Flu-infected WT and *Cars2*$^{+/-}$ mice. Scale bars, 100 µm. **j**, Representative images (left panel) and semi-quantification (right panel) of 8-OHdG immunostaining in lungs in Flu-infected WT mice given PBS or GSSSG (n = 3). Scale bars, 100 µm. Data are means ± s.d. $^{*}P < 0.05$, $^{***}P < 0.001$, $^{****}P < 0.0001$. Source data are provided as a Source Data file.

Supplementary Figure 3

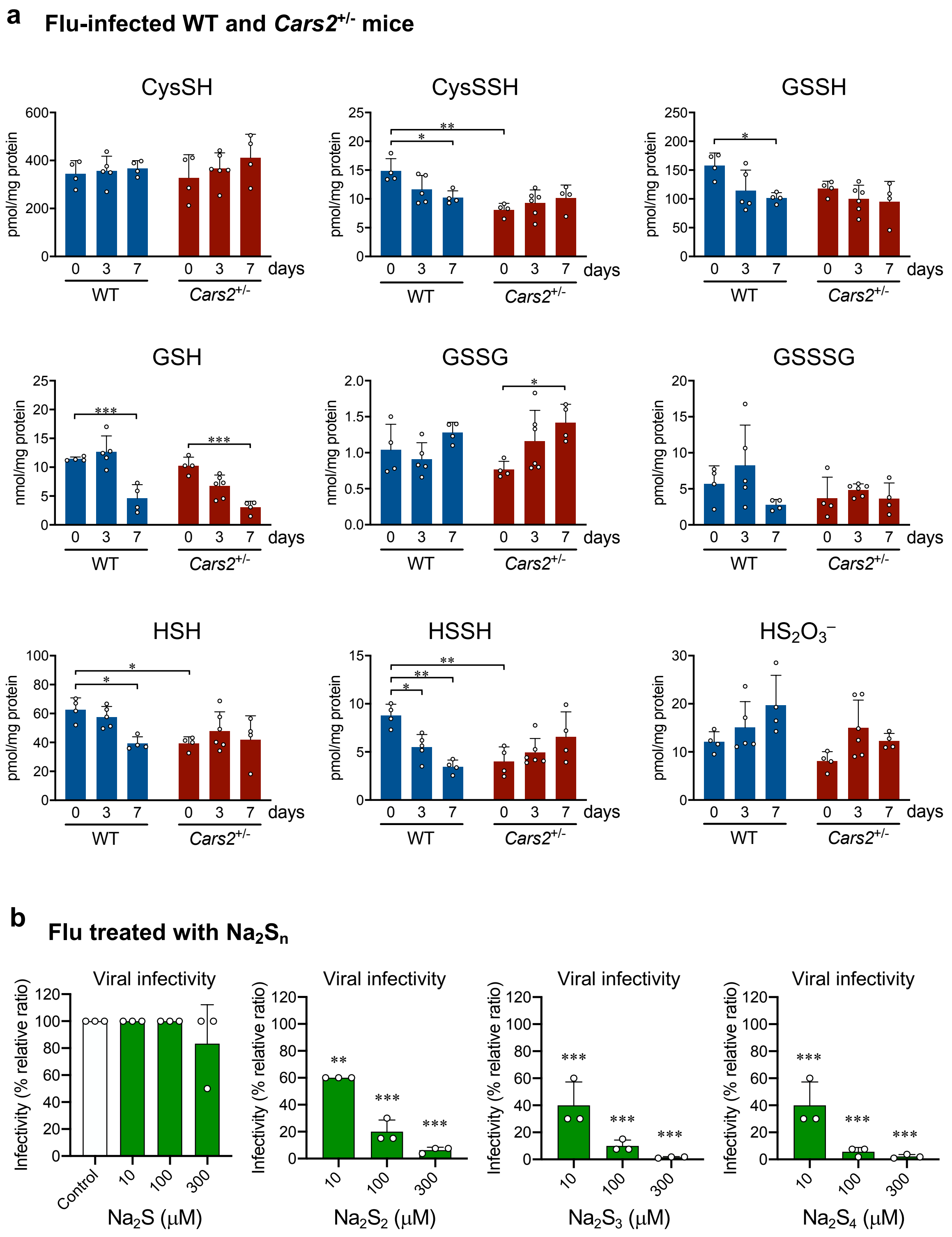

Supplementary Fig. 3 Supersulphide metabolome of influenza virus-infected mice lung (a) and attenuated influenza virus infectivity by supersulphides (b).

a, Production of supersulphides identified by means of LC-MS/MS analysis with HPE-IAM labeling in lungs obtained from WT and *Cars2*$^{+/-}$ littermates (*n* = 4-6). *P* = 0.042, 0.0015 (CysSSH), 0.049 (GSSH), 0.0006 (left in GSH), 0.0003 (right in GSH), 0.046 (GSSG), 0.040 (left and right in HSH), 0.048 (HSSH), 0.0011 (left in HSSH), and 0.0034 (right in HSSH). Data are means ± s.d. **P* < 0.05, ***P* < 0.01, ****P* < 0.001. **b,** Impairment of influenza virus infectivity by the treatment with supersulphides. After the influenza virus was incubated with 10, 100, and 300 μM $Na_2S_{1\text{-}4}$ at 37 ºC for 30 min, the virus with or without various Na_2S_n were inoculated to Madin-Darby canine kidney (MDCK) cells in culture. The viral infectivity was assessed by measuring the amount of infectious virus yielded in MDCK cells by the quantitative RT-PCR (*n* = 3). All *P* < 0.0001, but $^{**}P$ = 0.0013. $^{**}P$ < 0.01, $^{****}P$ < 0.0001 *vs*. control. Source data are provided as a Source Data file.

Supplementary Figure 4

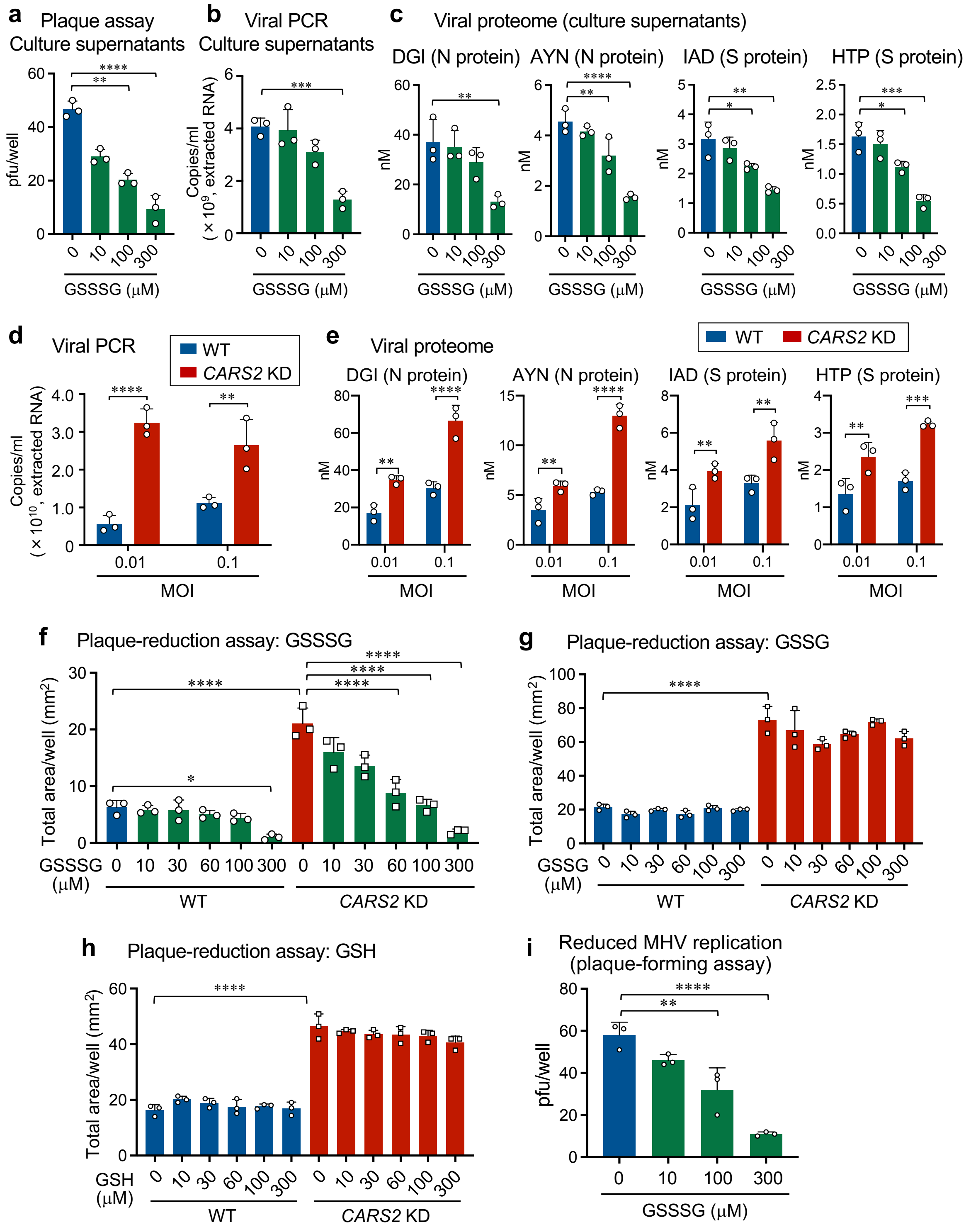

Supplementary Fig. 4 Anti-SARS-CoV-2 effects of supersulphides in VeroE6/TMPRSS2 cells with or without CARS2 KD.

a-c, The propagation of SARS-CoV-2 with or without various concentrations of GSSSG was

examined by the plaque-forming assay (on VeroE6/TMPRSS2 cells) **(a),** quantitative RT-PCR for the viral nucleoprotein RNA (**b**), and proteome analysis for the viral protein **(c),** with use of the culture supernatants harvested from the VeroE6/TMPRSS2 cells infected with SARS-CoV-2 at multiplicity of infection (MOI) of 0.01. *P* = 0.0028 and < 0.0001 in **a**. *P* = 0.0004 in **b**. *P* = 0.0078 (DGI), 0.015, < 0.0001 (AYN), = 0.045, 0.0014 (IAD), 0.020, and 0.0002 (HTP) in **c**. **d** and **e,** The effect of endogenous supersulphides was also examined by using VeroE6/TMPRSS2 cells with or without *CARS2* KD, which were infected with SARS-CoV-2 at MOI of 0.01 or 0.1. The yield of SARS-CoV-2 was then measured by the quantitative RT-PCR (**d)** and the proteome analysis (**e**) for the culture supernatants obtained from the parental (WT) and *CARS2* KD VeroE6/TMPRSS2 cells after SARS-CoV-2 infection. *P* < 0.0001 and = 0.0017 in **d**. *P* = 0.0031, < 0.0001 (DGI), = 0.0098, < 0.0001 (AYN), = 0.0076 (left in IAD), 0.0047 (right in IAD), 0.0041, and 0.0006 (HTP) in **e**. **f - h**, The plaque-reduction assay for GSSSG (**f**), GSSG (**g**), and GSH (**h**) with SARS-CoV-2 in WT and *CARS2* KD VeroE6/TMPRSS2 cells. All *P* < 0.0001, but **P* = 0.023 in **f**. *P* < 0.0001 in **g**. *P* < 0.0001 in **h**. **i**, Inhibitory effect of GSSSG on the replication of MHV. The reduced MHV replication in DBT cells treated with various concentrations of GSSSG was confirmed by measuring the MHV replication by the plaque-forming assay. *P* = 0.0023 and < 0.0001. Data are means ± s.d. (*n* = 3). $^{*}P < 0.05$, $^{**}P < 0.01$, $^{***}P < 0.001$, $^{****}P < 0.0001$. Source data are provided as a Source Data file.

Supplementary Figure 5

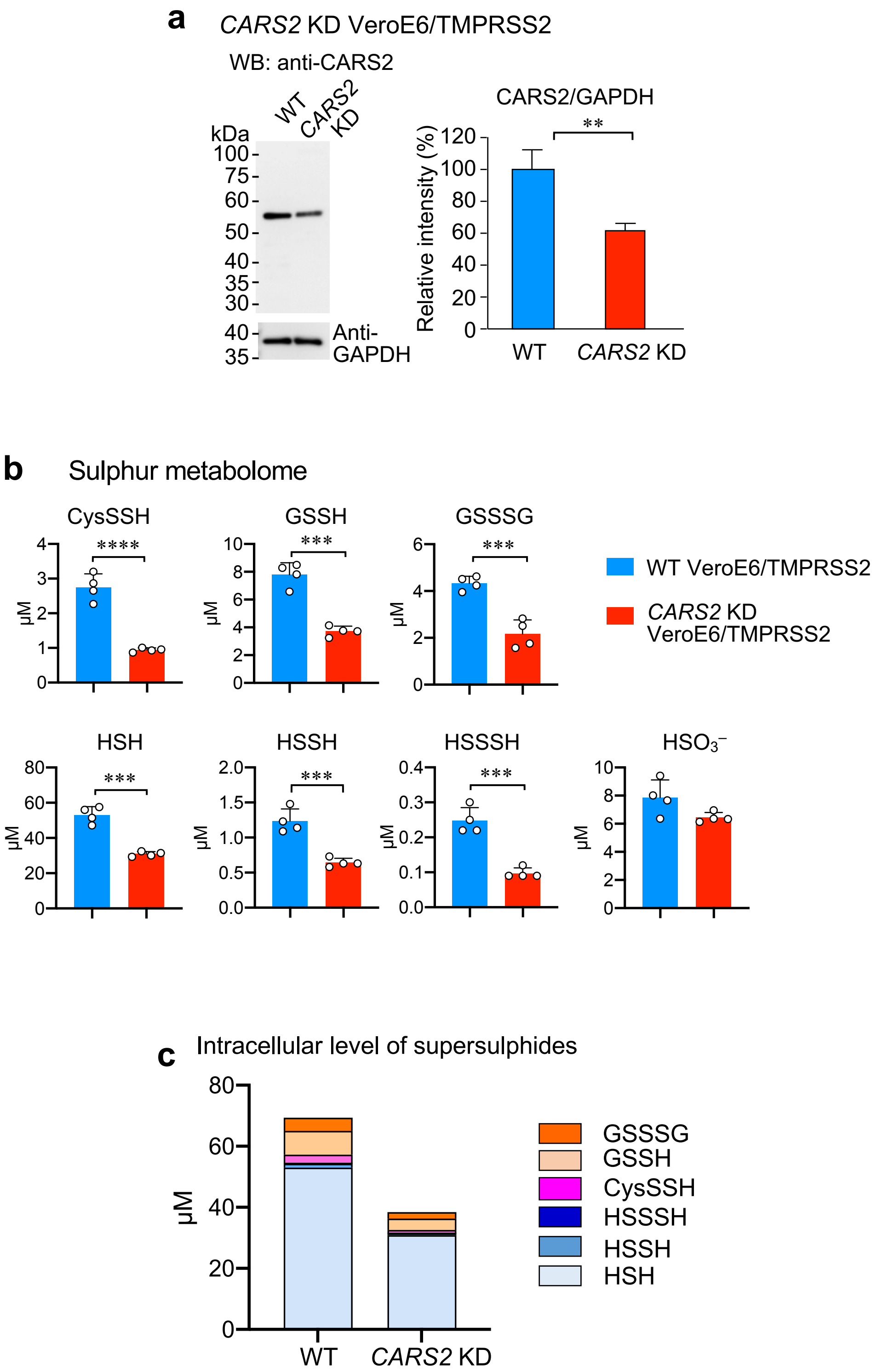

Supplementary Fig. 5 Endogenous production of supersulphides in WT and *CARS2* KD VeroE6/TMPRSS2 cells.

a, Generation of *CARS2* KD VeroE6/TMPRSS2 cells by using short hairpin RNAs (shRNA). The left and right panels show the CARS2 Western blotting and its densitometric data, respectively ($n = 3$). $P = 0.0013$. **b**, The supersulphide metabolome analysis for the *CARS2* KD VeroE6/TMPRSS2 cells ($n = 4$). $P < 0.0001$ (CysSSH), = 0.0001 (GSSH), 0.0007 (GSSSG), 0.0001 (HSH), 0.0007 (HSSH), 0.0003 (HSSSH). **c**, Total amounts of supersulphides quantified by the supersulphide metabolome with same the cells as in (**b**). Data are means ± s.d. $^{**}P < 0.01$, $^{***}P < 0.001$, $^{****}P < 0.0001$. Source data are provided as a Source Data file.

Supplementary Figure 6

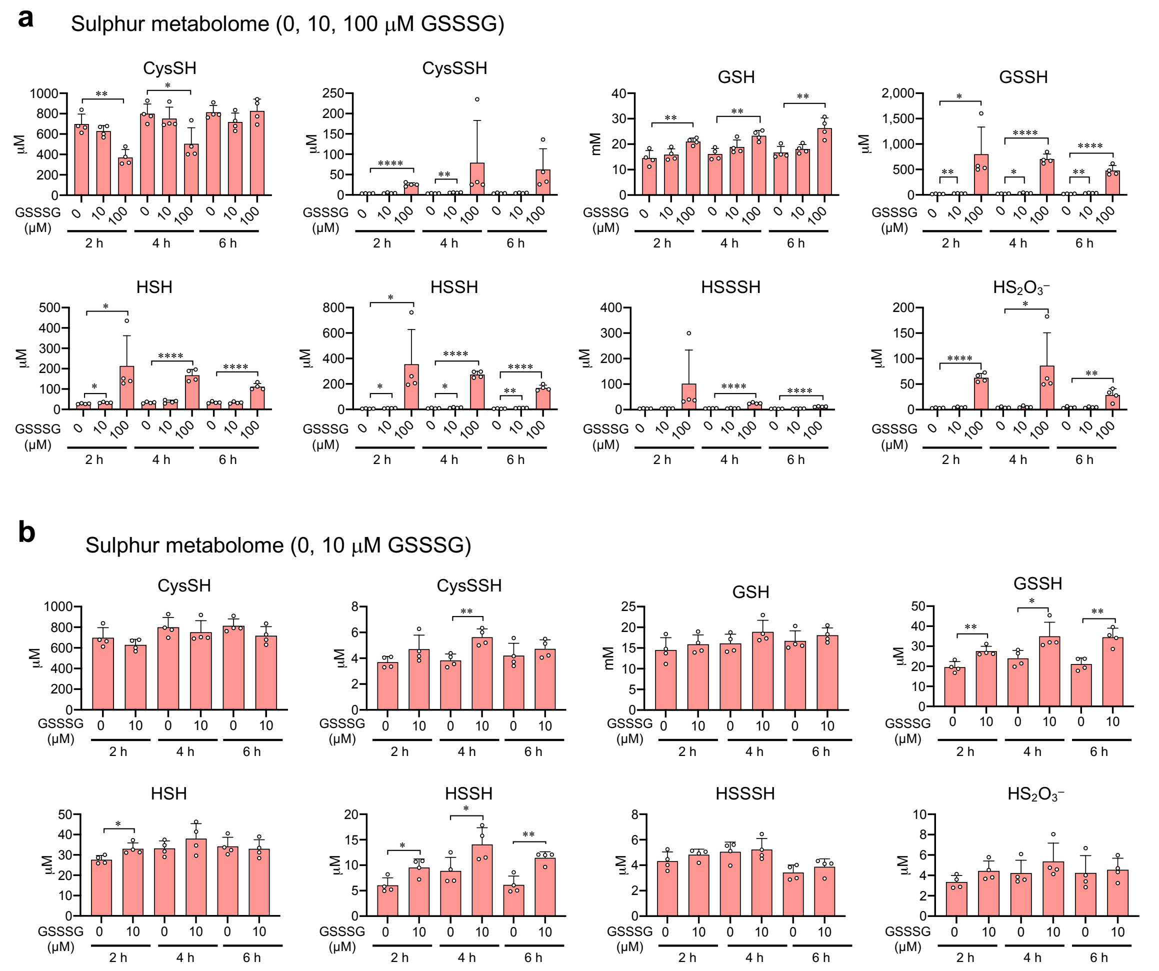

Supplementary Fig. 6 Supersulphides profiling of VeroE6/TMPRSS2 cells treated with GSSSG.

a, The supersulphide metabolome analysis with VeroE6/TMPRSS2 cells treated with various doses of GSSSG (0, 10, and 100 μM) for 2, 4, and 6 h. *P* values are described in **a**. **b**, The data obtained for the lower dose of GSSSG (10 μM) is highlighted by magnifying the vertical axis in (**a**). $P = 0.0044$ (CysSSH), 0.0048 (left in GSSH), 0.034 (GSSH), 0.0027 (right in GSSH), 0.022 (HSH), 0.020 (left in HSSH), 0.048 (right in HSSH), 0.0023 (HSSH) in **b**. Data are means ± s.d. ($n = 4$). $^{*}P < 0.05$, $^{**}P < 0.01$, $^{***}P < 0.001$, $^{****}P < 0.0001$. Source data are provided as a Source Data file.

Supplementary Figure 7

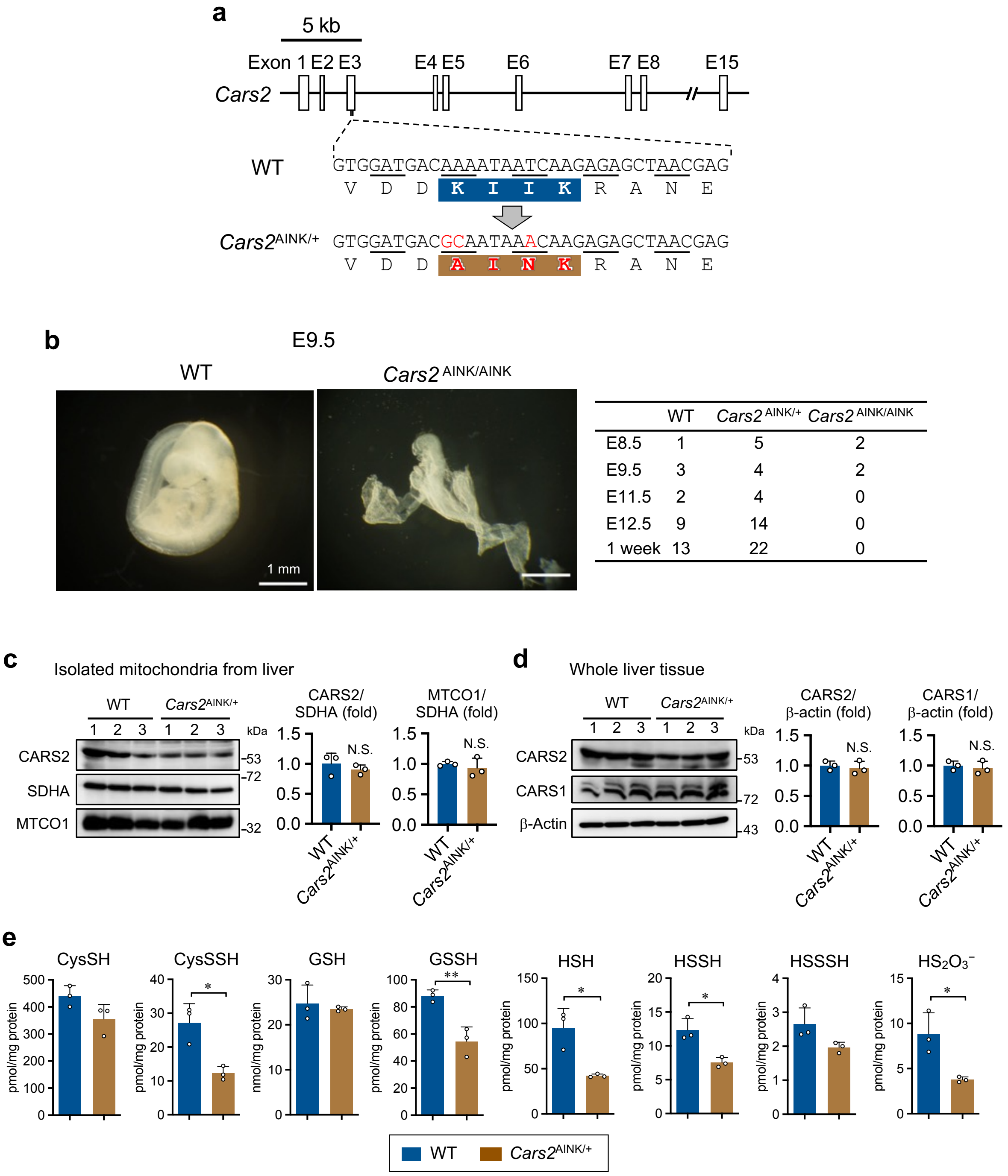

Supplementary Fig. 7 Generation of CPERS activity-selective *Cars2*-mutant mice (*Cars2*$^{\mathrm{AINK/+}}$).

a, Generation of heterozygous of *Cars2*-mutant (*Cars2*$^{\mathrm{AINK/+}}$) mice with defective CPERS activity but with intact Cys-tRNA synthetase activity. Schematic illustration of the *Cars2* gene structure and sequences of WT and mutant alleles surrounding KIIK motif.

Nucleotides marked in red indicate mutations induced by genome editing. **b**, Representative gross morphology of WT and homozygous *Cars2*$^{AINK/AINK}$ littermate embryos at embryonic day (E) 9.5. *Cars2*$^{AINK/AINK}$ embryos exhibit apparent growth retardation at E9.5. Scale bars indicate 1 mm. Right panel: the genotyping results of 46 embryos and 35 pups in the mating of *Cars2*$^{AINK/+}$ mice. No *Cars2*$^{AINK/AINK}$ embryos were observed after Embryonic day 11.5, suggesting that *Cars2*$^{AINK/AINK}$ embryos are lethal at midgestation stage. Scale bars, 1 mm. **c** and **d**, Levels of CARS2 protein in isolated liver mitochondria (left panel) and in whole liver tissue (right panel) from WT and *Cars2*$^{AINK/+}$ mice as determined by using the Western blotting; quantitative results appear in lower panels. **e**, In vivo formation of supersulphides in WT and *Cars2*$^{AINK/+}$ mice. Endogenous production of CysSSH and other related supersulphide metabolites in lungs obtained from WT mice and *Cars2*$^{AINK/+}$ littermates were quantified via LC-MS/MS analysis with HPE-IAM labeling. P = 0.012 (CysSSH), 0.0069 (GSSH), 0.012 (HSH), 0.011 (HSSH), and 0.021 ($HS_2O_3^-$). Data are means ± s.d. (n = 3). $^{*}P < 0.05$, $^{**}P < 0.01$, N.S., not significant. Source data are provided as a Source Data file.

Supplementary Figure 8

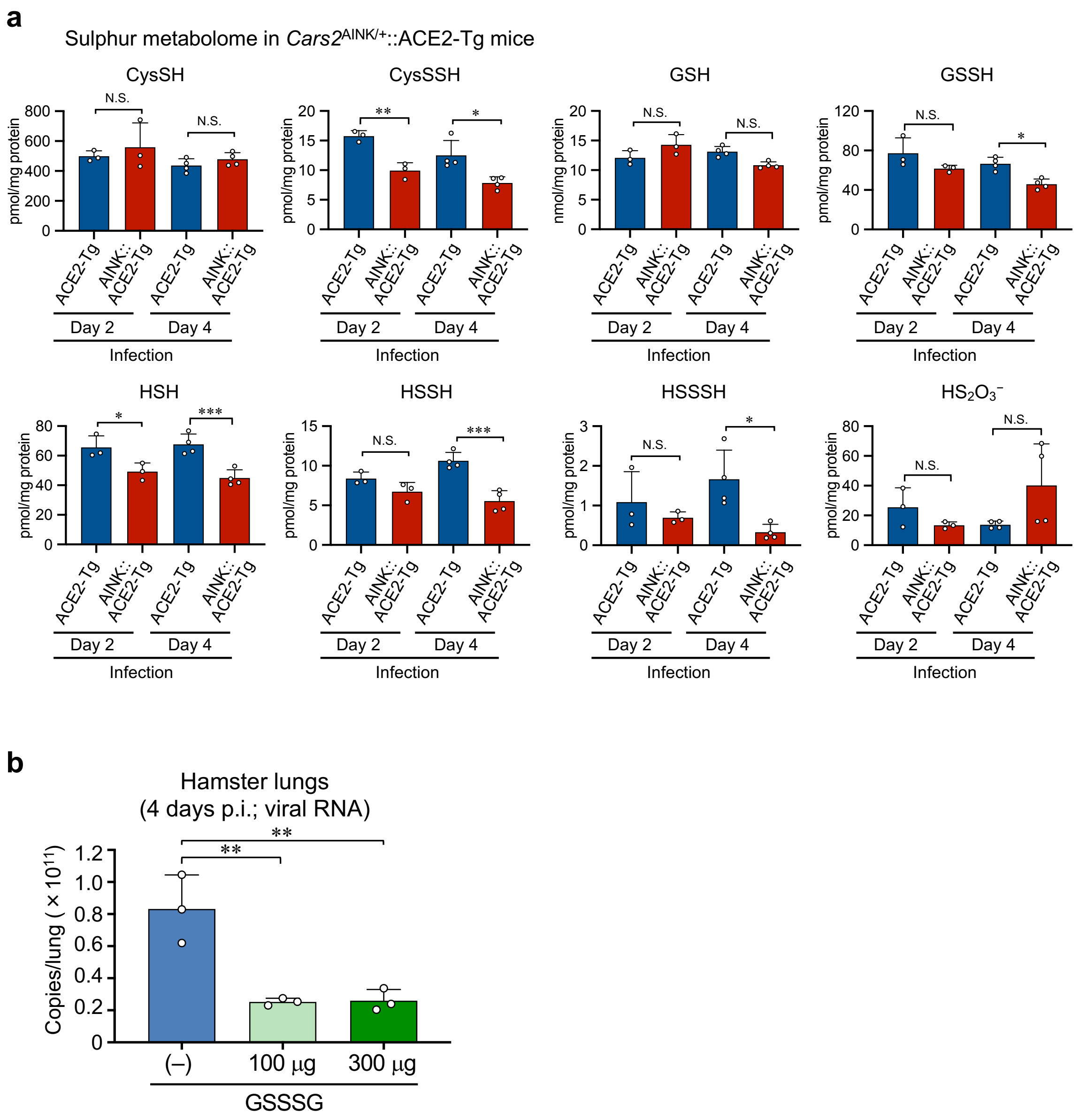

Supplementary Fig. 8 Supersulphide metabolome with SARS-CoV-2-infected ACE2-AINK-mutant mice (a) and SARS-CoV-2 yielded in the hamster lungs (b).

a, In vivo formation of supersulphides in the infected *Cars2*$^{AINK/+}$::ACE2-Tg (AINK::ACE2-Tg) mice. All mice were i.t. infected with 100 pfu (50 μl) of SARS-CoV-2. Supersulphides production in lungs of the ACE2-Tg and AINK::ACE2-Tg mice at 2 and 4 days post-infection (LC-MS/MS analysis with HPE-IAM labeling). P = 0.0073, 0.013 (CysSSH), 0.027 (GSSH), 0.044, 0.0030 (HSH), 0.0004 (HSSH), and 0.026 (HSSSH). **b**, The amounts of virus yielded

in the lungs of hamsters treated or untreated with GSSSG after infection, as assessed by quantitative RT-PCR at 4 days post-infection. $P = 0.0038$ (left) and 0.0041 (right). Data are means ± s.d. ($n = 3$-4). $^{*}P < 0.05$, $^{**}P < 0.01$, $^{***}P < 0.001$, N.S., not significant. Source data are provided as a Source Data file.

Supplementary Figure 9

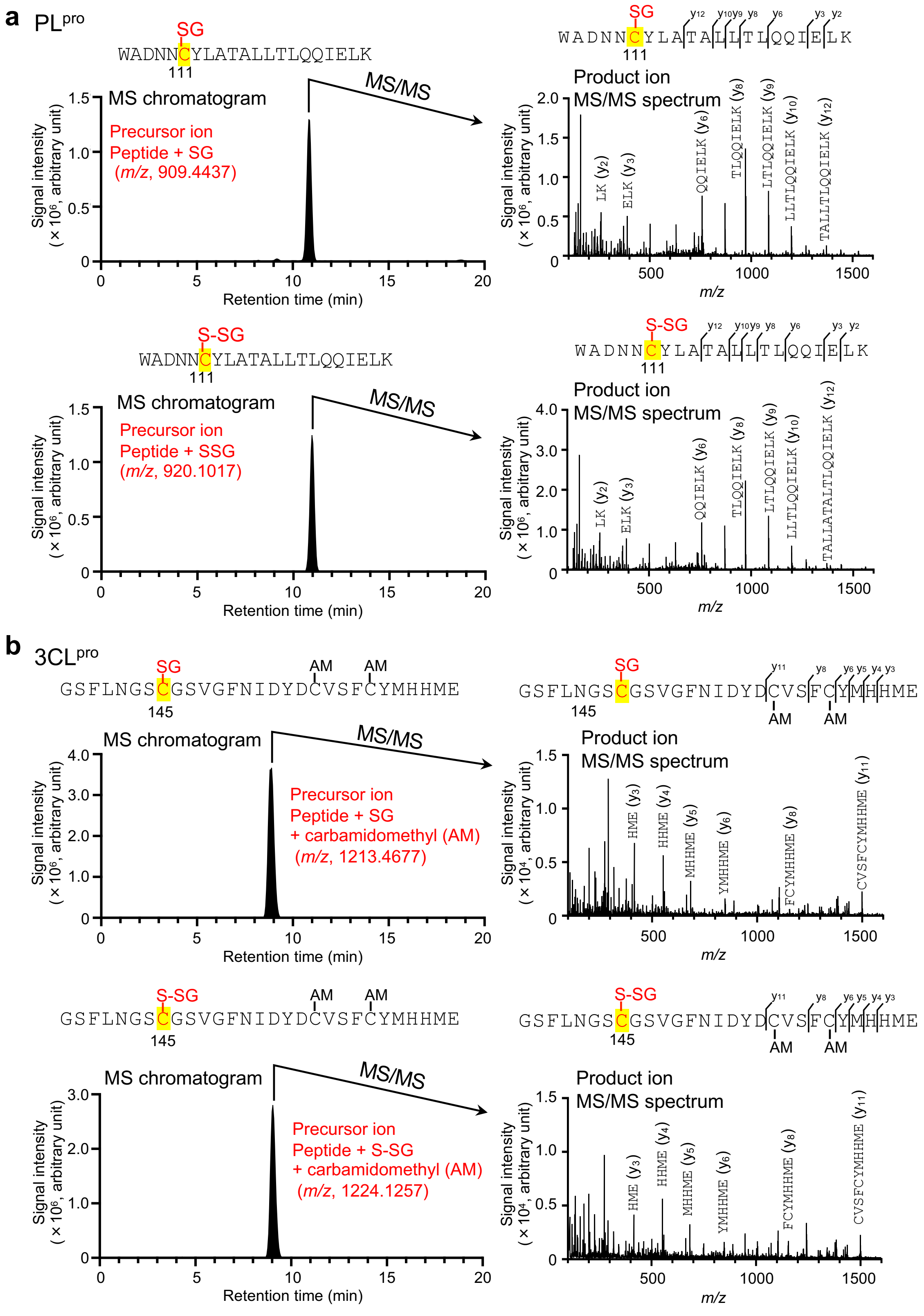

Supplementary Fig. 9 Proteome analysis of GSSSG-treated PL^{pro} and 3CL^{pro} using LC-

ESI-Q-TOF-MS/MS.

a, PL^{pro} proteome: LC-Q-TOF-MS chromatograms of PL^{pro} peptide fragments which including cysteine residue (C111) [glutathiloation (left upper panel) and perthioglutathionylation (left lower panel)]. MS/MS product ion search in detected glutathiolated (right upper panel) and perthioglutathionylated peptides (right lower panel). **b**, $3CL^{pro}$ proteome: LC-Q-TOF-MS chromatograms of $3CL^{pro}$ peptide fragments which including cysteine residue (C145) [glutathionylation (left upper panel) and perthioglutathionylation (left lower panel)]. MS/MS product ion search in detected glutathionylated (right upper panel) and perthioglutathionylated peptides (right lower panel). PL^{pro} and $3CL^{pro}$ incubated with GSSSG were alkylated and digested, followed then subjected to proteome analysis. Glutathionylation of cysteine residues in PL^{pro} and $3CL^{pro}$ were detected by monitoring at *m/z* 909.4437 and 1213.4677, respectively. Perthioglutathionylation of cysteine residues in PL^{pro} and $3CL^{pro}$ were detected by monitoring at *m/z* 920.1017 and 1224.1257, respectively. Source data are provided as a Source Data file.

Supplementary Figure 10

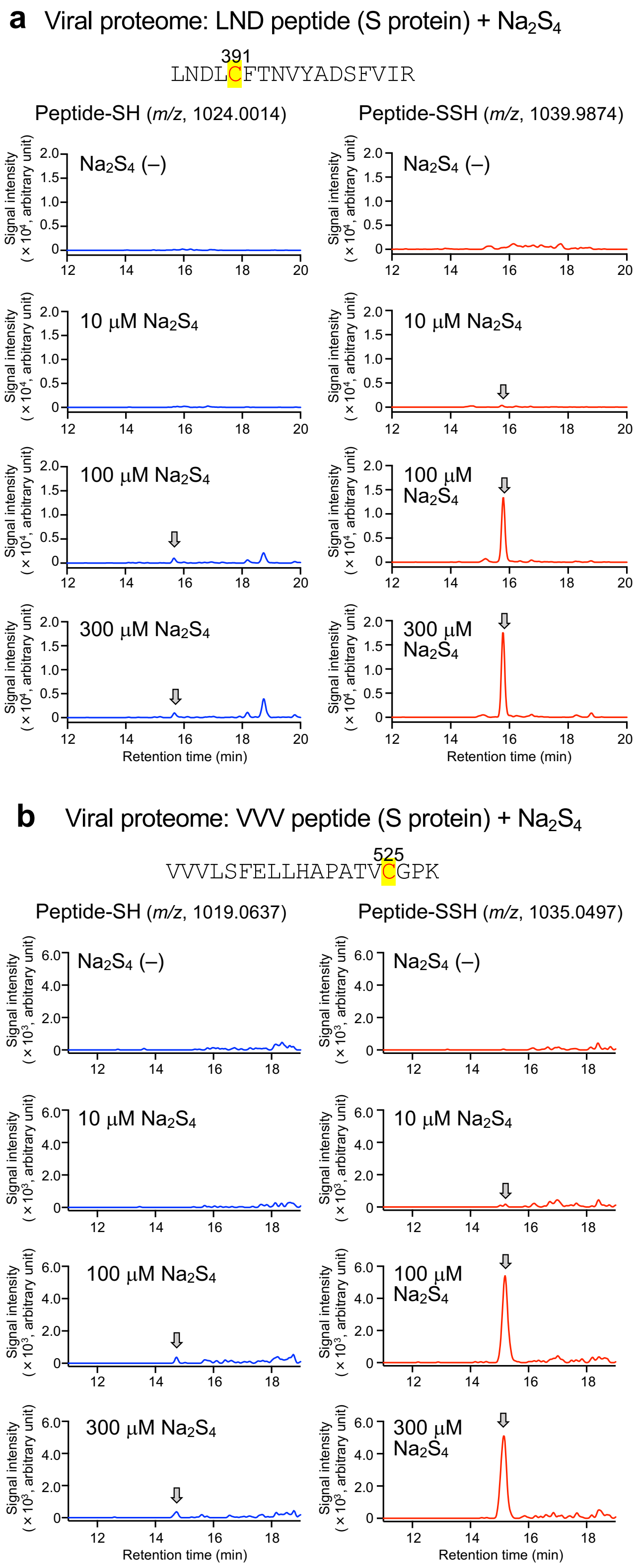

Supplementary Fig. 10 Proteome analysis of cysteine residues in spike protein using LC-

ESI-Q-TOF/MS.

LC-ESI-Q-TOF-MS chromatograms via the proteome analysis of peptide fragments from the spike (S) protein, which include cysteine residues (C391, **a** and C525, **b**). S proteins that were reacted with Na_2S_4 (0-300 μM), were subjected to the MS analysis. Polysulphides of C391 and C525 in S protein were detected by monitoring at *m/z* 1024.0014 and 1019.0637 for CysS-AM, *m/z* 1039.9874 and 1035.0497 for CysS-S-AM, and *m/z* 1055.9734 and 1051.0357 for CysS-SS-AM, respectively. Source data are provided as a Source Data file.

Supplementary Figure 11

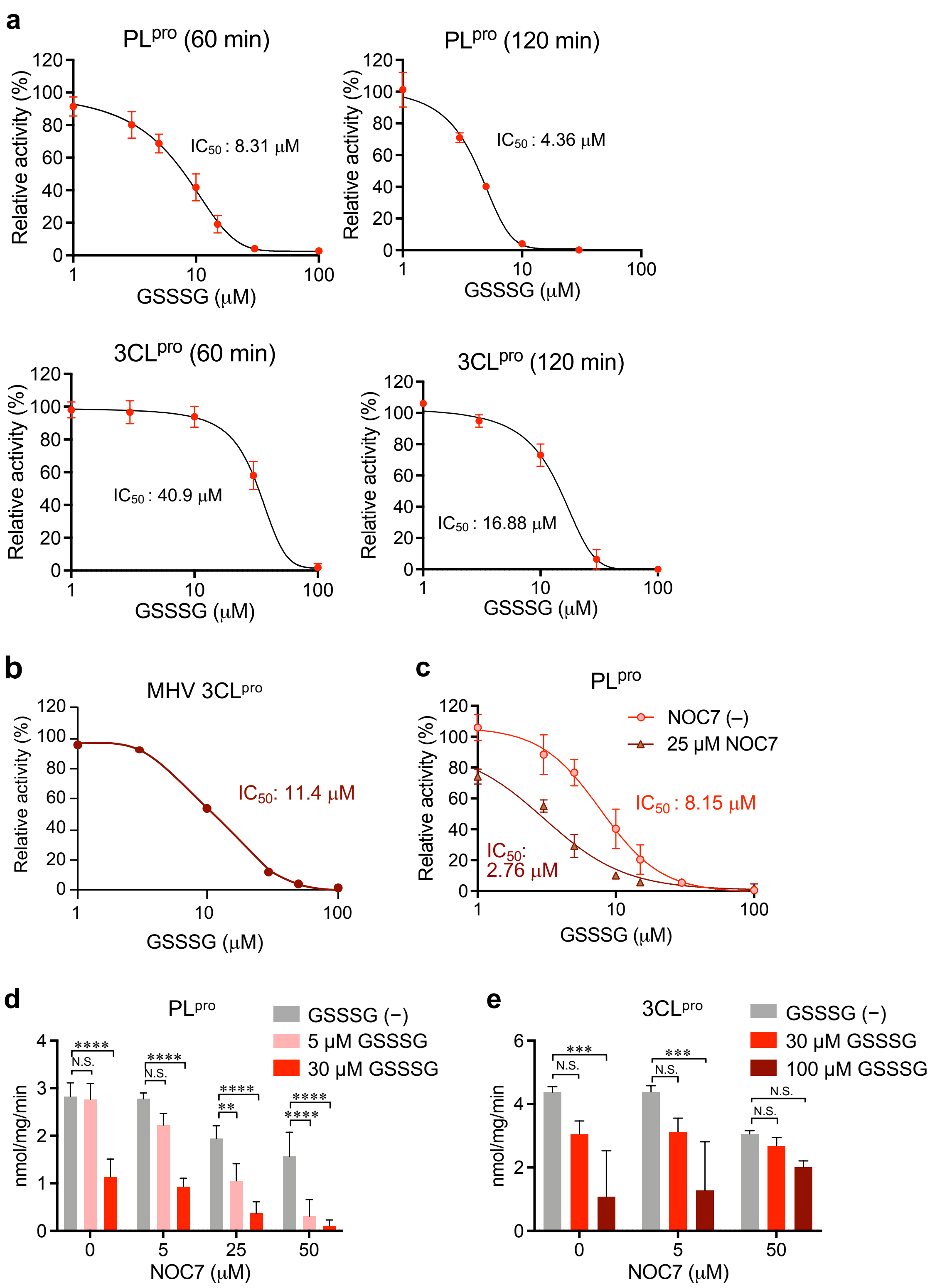

Supplementary Fig. 11 Inhibitory effect of GSSSG on the SARS-CoV-2 thiol proteases

PL^{pro} and $3CL^{pro}$.

a, Inhibitory profiles of GSSSG on PL^{pro} (upper panels) and $3CL^{pro}$ activity (lower panels) at 60 min (left panels) and 120 min (right panels). PL^{pro} or $3CL^{pro}$ at 1 μM each was reacted with various concentrations of GSSSG, followed by measuring the proteases activity. Dose-response curves for the IC_{50} values were obtained by using nonlinear regression. **b**, Inhibitory effect of GSSSG on $3CL^{pro}$ activity of MHV (1 μM). Dose-response curves for half-maximal inhibitory concentration (IC_{50}) values were calculated by using nonlinear regression. **c-e**, Enhancing effect of NO on inhibitory potential of GSSSG against PL^{pro}. PL^{pro} (3 μM) was treated with various concentrations of NOC7 (**c**, 25 μM) as an NO donor, after that PL^{pro} at 1 μM was reacted with various concentrations of GSSSG, followed by measurement of protease activity. Dose-response curves for IC_{50} values were calculated by using nonlinear regression. All $P < 0.0001$, but $^{**}P = 0.0037$ in **d**. $P = 0.0001$ (left) and 0.0002 (right) in **e**. Data are means ± s.d. ($n = 3$). $^{**}P < 0.01$, $^{***}P < 0.001$, $^{****}P < 0.0001$, N.S., not significant. Source data are provided as a Source Data file.

Supplementary Figure 12

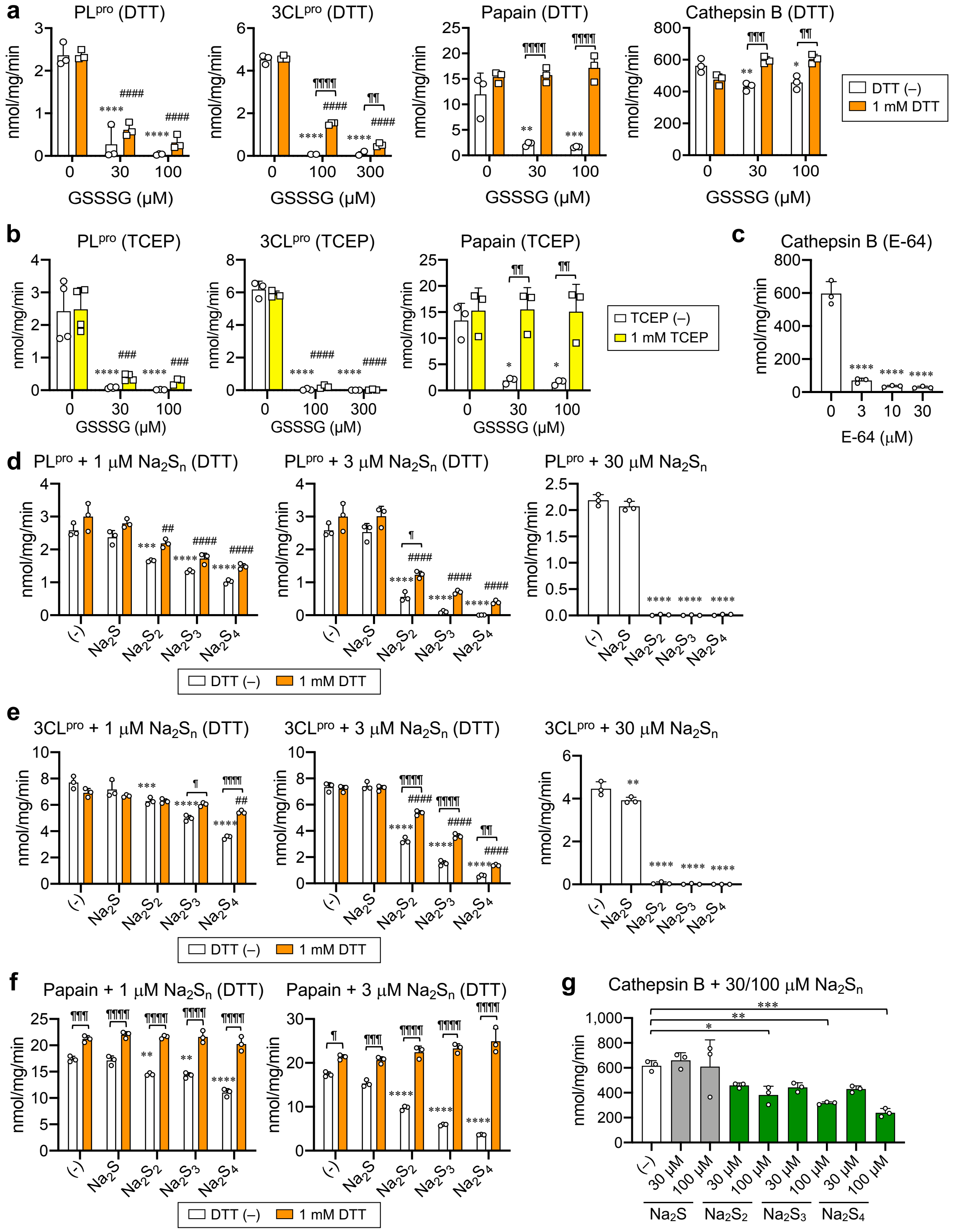

Supplementary Fig. 12 Inhibitory profiles of supersulphides on the SARS-CoV-2 PL^{pro} and 3CL^{pro}.

a, Inhibitory effect of GSSSG on PL^{pro}, 3CL^{pro}, papain, and cathepsin B. The protease activity was examined in terms of the effect of reductive treatment with dithiothreitol (DTT)

after GSSSG treatment of the proteases. All $P < 0.0001$, but $^{\P\P}P = 0.0099$ ($3CL^{pro}$, DTT), $^{**}P = 0.0014$, $^{***}P = 0.0008$ (Papain, DTT), $^{*}P = 0.026$, $^{**}P = 0.0056$, $^{\P\P}P = 0.0022$, and $^{\P\P\P}P =$ 0.0008 (Cathepsin B, DTT). Data are means ± s.d. ($n = 3$). $^{*}P < 0.05$, $^{**}P < 0.01$, $^{***}P <$ 0.001, $^{****}P < 0.0001$ *vs.* 0 μM GSSSG/DTT (–); $^{\#\#\#\#}P < 0.0001$ *vs.* 0 μM GSSSG/1 mM DTT; $^{\P\P}P < 0.01$, $^{\P\P\P}P < 0.001$, $^{\P\P\P\P}P < 0.0001$. **b**, Inhibitory effect of GSSSG on PL^{pro}, $3CL^{pro}$, papain, and cathepsin B. The protease activity was examined in terms of the effect of reductive treatment with tris(2-carboxyethyl)phosphine (TCEP) after GSSSG treatment of the proteases. All $P < 0.0001$, but $^{\#\#\#}P = 0.0002$ (left and right in PL^{pro}, TCEP), $^{*}P = 0.017$ (left in papain, TCEP), $^{*}P = 0.014$ (right in papain, TCEP), $^{\P\P}P = 0.0050$ (left in papain, TCEP), and $^{\P\P}P = 0.0052$ (right in papain, TCEP). Data are means ± s.d. ($n = 3$). $^{***}P < 0.001$, $^{****}P$ < 0.0001 *vs.* 0 μM GSSSG/TCEP (–); $^{\#\#\#}P < 0.001$, $^{\#\#\#\#}P < 0.0001$ *vs.* 0 μM GSSSG/1 mM TCEP; $^{\P\P}P < 0.01$. **c**, Cathepsin B activity was inhibited by a specific thiol protease inhibitor, E-64. All $P < 0.0001$. Data are means ± s.d. ($n = 3$). $^{****}P < 0.0001$ *vs.* 0 μM E-64. **d** and **e**, Inhibitory effect of 1, 3, and 30 μM $Na_2S_{1\text{-}4}$ on PL^{pro} (**d**) or $3CL^{pro}$ (**e**). The protease activity was examined in terms of the effect of reductive treatment with DTT after Na_2S_n treatment of the proteases. All $P < 0.0001$, but $^{***}P = 0.0002$, $^{\#\#}P = 0.0012$, $^{\P}P = 0.030$ in **d**. All $P < 0.0001$, but $^{***}P = 0.0009$, $^{\P}P = 0.025$, $^{\#\#}P = 0.0053$, $^{\P\P}P = 0.0095$, $^{**}P = 0.0066$ in **e**. Data are means ± s.d. ($n = 3$). $^{***}P < 0.001$, $^{****}P < 0.0001$ *vs.* 0 μM Na_2S_n/DTT (–); $^{\#\#}P <$ 0.01, $^{\#\#\#\#}P < 0.0001$ *vs.* 0 μM Na_2S_n /1 mM DTT; $^{\P}P < 0.05$ in **d**. $^{***}P < 0.001$, $^{****}P < 0.0001$ *vs.* 0 μM Na_2S_n/DTT (–); $^{\#\#}P < 0.01$, $^{\#\#\#\#}P < 0.0001$ *vs.* 0 μM Na_2S_n /1 mM DTT; $^{\P}P < 0.05$, $^{\P\P}P < 0.01$, $^{\P\P\P\P}P < 0.0001$ in **e**. **f**, Inhibitory effect of 1 and 3 μM $Na_2S_{1\text{-}4}$ on papain in terms of the effect of reductive treatment with DTT. All $P < 0.0001$, but $^{\P\P\P}P = 0.0003$ [papain, 1 μM Na_2S_n(-)], $^{**}P = 0.0089$ [papain, 1 μM Na_2S_2, DTT(-)], $^{**}P = 0.0043$ [papain, 1 μM Na_2S_3, DTT(-)], $^{\P}P = 0.022$, $^{\P\P\P}P = 0.0007$ (papain, 3 μM Na_2S) in **f**. Data are means ± s.d. ($n = 3$). $^{**}P < 0.01$, $^{****}P < 0.0001$ *vs.* 0 μM Na_2S_n/DTT (–); $^{\P}P < 0.05$, $^{\P\P\P}P < 0.001$, $^{\P\P\P\P}P < 0.0001$. **g**, Inhibitory effect of 30 and 100 μM $Na_2S_{1\text{-}4}$ on cathepsin B in terms of the effect of reductive treatment with DTT. $P = 0.019$, 0.0022, and 0.0002. Data are means ± s.d. ($n = 3$). $^{*}P <$ 0.05, $^{**}P < 0.01$, $^{***}P < 0.001$.

Cathepsin B (a major endogenous thiol protease) looks like less susceptible to supersulphides compared to the viral thiol proteases. Supersulphides may thus modify and inactivate selectively the viral thiol proteases and other structural proteins. On the contrary, supersulphides only moderately affect the host's proteins containing critical thiols and

disulphides, suggesting a physiologically relevant host defense that could be mediated by supersuphides against viruses and many other pathogens. Source data are provided as a Source Data file.

Supplementary Figure 13

a

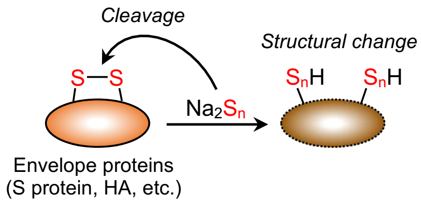

b

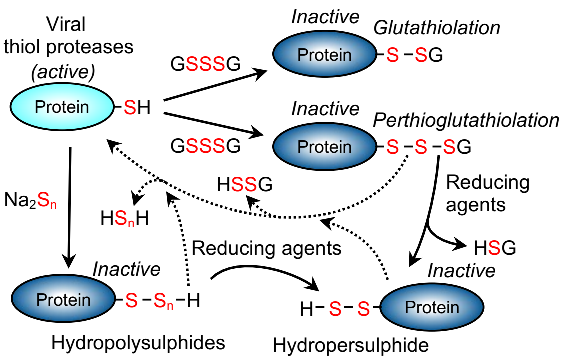

Supplementary Fig. 13 Antiviral mechanisms through supersulphide-mediated thiol modifications.

a, Schematic drawing of the reaction of viral envelope proteins with inorganic polysulfides. The inorganic polysulphide donors Na_2S_{2-4} target not only cysteine thiol residues of viral cysteine proteases but also cysteines disulphide bridges that are formed in viral envelope proteins such as the spike protein of SARS-CoV-2 and HA of influenza virus. Upon treatment with Na_2S_{2-4}, the disulphide formation of envelope proteins is cleaved off, which leads to conformational alteration of the proteins and thereby elimination of viral infectivity to infect host cells. In this context, we identified herein the appreciable amounts of inorganic hydropolysulphides not only in cultured cells but also in vivo in mouse lungs and even in the breath aerosol exhaled from the airways of human and hamsters, which significantly increased after SARS-CoV-2 infections (see Fig. 5). Therefore, the direct antiviral effect of supersulphides on viral particles per se is thought to be physiological relevant to the antiviral host defense.

b, Schematic illustration of the reaction of SARS-CoV-2 thiol proteases with GSSSG and $Na_2S_{1\text{-}4}$. GSSSG inhibits the viral thiol protease by reacting with the cysteine residues at the active centres of the thiol proteases, through glutathionylationor perthioglutathionylation of cysteine. Glutathiolated or perthioglutathiolated cysteine residues can be reduced by reducing agents such as DTT or TCEP to yield thiol and cysteine persulphide residues. The activity is restored upon reduction of the thiol residues, while the hydropersulphide (RSSH) residue is partially resistant to reducing agents and the protease activity thereby remains inactivated, as shown in this study (Supplementary Fig. 12). In fact, inorganic polysulphides ($Na_2S_{2\text{-}4}$) inactivate the protease activity by modifying the active site cysteine residues to the corresponding hydropolysulphides, which are also resistant to the reducing agents in terms of reactivation of the protease activity. The reductive reaction pathways (indicated with dashed lines) returning to the original simple thiol (RSH) of viral proteins may not be feasible because of resistance of their RS_nH residues to reducing agents, as schematized herein.

Supplementary Figure 14

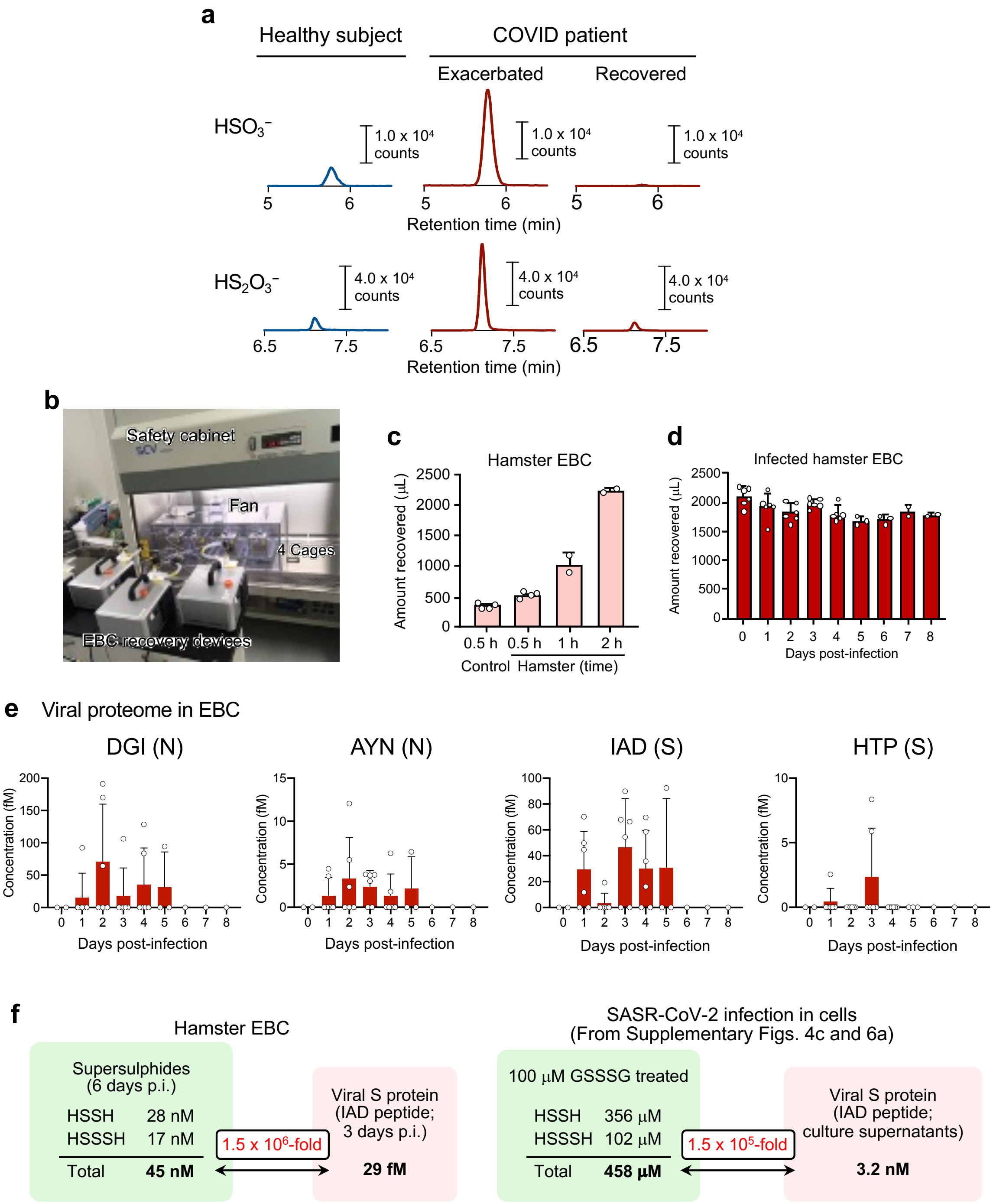

Supplementary Fig. 14 Breath omics analyses in human and hamsters (a-e) and comparison of supersulphides *vs.* SARS-CoV-2 yielded in EBC or cultured cells (f).

a, Representative LC-MS/MS chromatograms obtained by analysis of HSO_3^-, and $HS_2O_3^-$ formed in EBC of a healthy subject and COVID-19 patient. These oxidized sulphides may be produced from supersulphides through their regulatory and scavenging reactions with

reactive oxygen species, which further suggests that the oxidative stress response mediated by supersulphides may be a hallmark of the COVID-19 pathogenesis. **b**, The hamster EBC collection system. Safety cabinet, fan, cages, and EBC recovery device are shown. **c**, Amount of hamster EBC recovered depends on the recovery time. **d**, Amount of hamster EBC recovered are not significantly altered during the infection protocol for 8 days post-infection. **e**, Viral proteome analysis in infected hamster EBC during 8 days post-infection detected by LC-ESI-MS/MS. **f**, Quantitative comparison of sulphide metabolites and viral spike proteins in hamster EBC. In terms of the antiviral host defense, it is noteworthy that the relative amounts of supersuphides *vs*. SARS-CoV-2 observed in the EBC (1.5×10^6-fold: Figs. 5d-f), are found in a much higher range than that of their relative amounts (1.5×10^5-fold) to get appreciable antiviral effects on SARS-CoV-2 propagating in VeroE6/TMPRSS2 cells (10^5-fold: Supplementary Figs. 4c, 6a). Data are means ± s.d. Source data are provided as a Source Data file.

Supplementary Figure 15

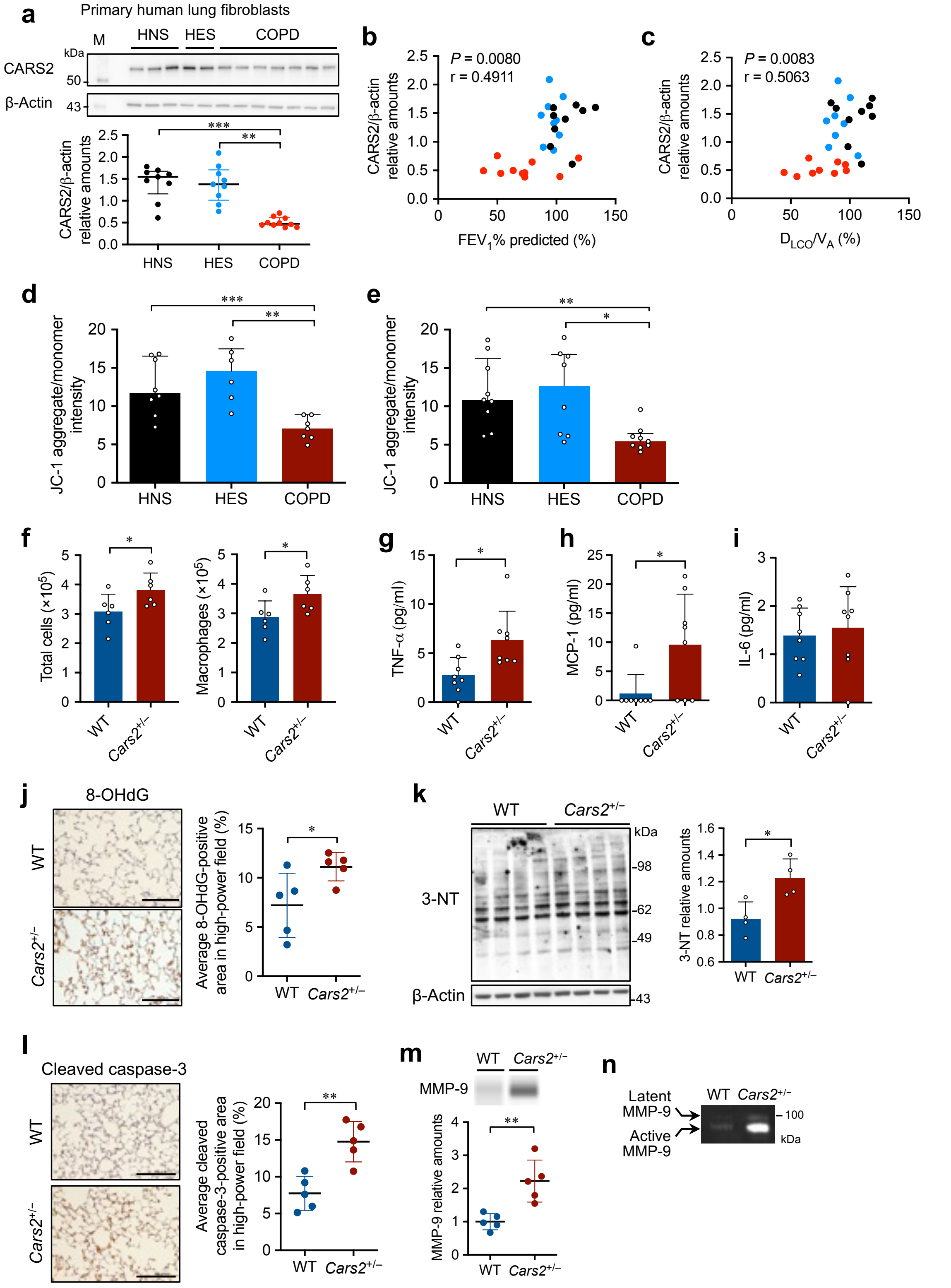

Supplementary Fig. 15 Reduced CARS2 in primary lung-resident cells from patients with COPD and enhanced elastase-induced airway inflammation and senescence in

lungs of *Cars2*$^{+/-}$ mice.

a, CARS2 expression in primary lung fibroblasts from patients with COPD (n = 10) and from HNS (n = 9) and HES (n = 9) as determined by Western blotting (upper panel) and quantitative results (lower panel). β-Actin was used as the loading control. P = 0.0006 and 0.0013. **b** and **c**, Correlations between the amounts of CARS2 and FEV_1% predicted (**b**) or D_{LCO}/V_A predicted (**c**) of study subjects. Black, blue, and red circles indicate HNS, HES, and COPD, respectively. **d** and **e**, Quantification of mitochondrial membrane potential, as measured by using JC-1, in primary bronchial epithelial cells (**d**) and primary lung fibroblasts (**e**) from HNS, HES, and patients with COPD. Each dot represents data from an individual subject (n = 6-9 per group). P = 0.030 and 0.0045 in **d**. P = 0.0062 and 0.020 in **e**. **f**, Numbers of total cells and macrophages in BALF obtained from WT and *Cars2*$^{+/-}$ mice (n = 6) on day 5 after treatment with porcine pancreatic elastase (PPE). P = 0.030 (left) and 0.046 (right). **g-i**, Measurement of TNF-α (**g**), MCP-1 (**h**), and IL-6 (**i**) in BALF obtained on day 5 by using a cytometric bead array (n = 8). P = 0.011 in **g**. P = 0.023 in **h**. **j,** Representative immunohistochemistry for 8-OHdG in lung sections obtained from WT and *Cars2*$^{+/-}$ mice (n = 5) treated with PPE on day 5. Immunopositive areas were semi-quantified by using ImageJ. P = 0.039. Scale bars, 100 μm. **k**, Formation of 3-NT in lung homogenates obtained from WT and *Cars2*$^{+/-}$ mice (n = 4) treated with PPE on day 5 as determined by means of Western blotting. β-Actin was used as the loading control. P = 0.018. **l**, Representative immunohistochemistry for cleaved caspase-3 in lung sections obtained from WT and *Cars2*$^{+/-}$ mice (n = 5) treated with PPE on day 5. Immunopositive areas were semi-quantified by using ImageJ. P = 0.0024. Scale bars, 100 μm. **m**, Amounts of total matrix metalloproteinase-9 (MMP-9) in BALF from WT and *Cars2*$^{+/-}$ mice (n = 5) as determined by the capillary Western blot analysis (Simple Western™). P = 0.0038. **n**, Representative image of gelatinase activity of MMP-9 in BALF as determined by means of gelatin zymography. BALF was obtained from mice treated with PPE on day 5. Data are means ± s.d. $^{*}P < 0.05$, $^{**}P < 0.01$. Source data are provided as a Source Data file.

Supplementary Figure 16

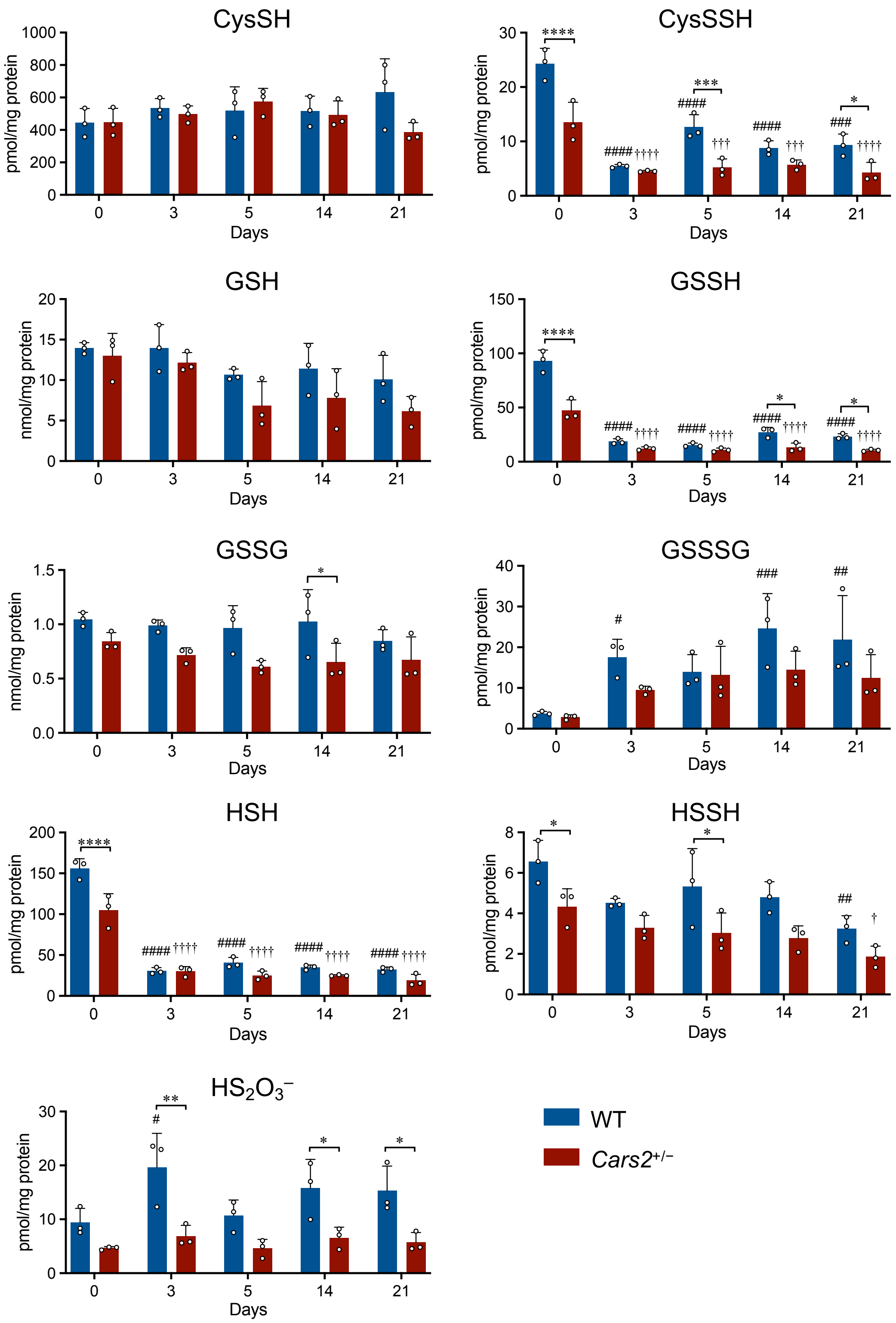

Supplementary Fig. 16 Supersulphide metabolome in lungs of WT and *Cars2*$^{+/-}$ mice

treated with elastase.

Production of supersulphide in lungs obtained from elastase-treated WT and *Cars2*$^{+/-}$ littermates was determined by means of LC-MS/MS analysis with HPE-IAM labeling. Data are means ± s.d. Each dot represents data from an individual mouse (n = 3 per group). All P < 0.0001, but $^{***}P$ = 0.0009, $^{†††}P$ = 0.0002 (*Cars2*$^{+/-}$, 5 days), $^{†††}P$ =0.0004 (*Cars2*$^{+/-}$, 14 days), $^{*}P$ = 0.029 in CysSSH. All P < 0.0001, but $^{*}P$ = 0.014 (14 days), $^{*}P$ = 0.029 (21 days) in GSSH. P = 0.039 in GSSG. P = 0.021, 0.0008, 0.0028 in GSSSG. All P < 0.0001 in HSH. $^{*}P$ = 0.037 (0 day), $^{*}P$ = 0.032 (5 days), $^{\#\#}P$ = 0.0016, $^{†}P$ = 0.016 in HSSH. $^{\#}P$ = 0.016, $^{**}P$ = 0.0010, $^{*}P$ = 0.019 (14 days), $^{*}P$ = 0.014 (21 days) in $HS_2O_3^-$. $^{*}P$ < 0.05, $^{**}P$ < 0.01, $^{***}P$ < 0.001, $^{****}P$ < 0.0001; $^{\#}P$ < 0.05, $^{\#\#}P$ < 0.01, $^{\#\#\#}P$ < 0.001, $^{\#\#\#\#}P$ < 0.0001 *vs*. WT mice at day 0; $^{†}P$ < 0.05, $^{††}P$ < 0.01, $^{†††}P$ < 0.001, $^{††††}P$ < 0.0001 *vs*. *Cars2*$^{+/–}$ mice at day 0. Source data are provided as a Source Data file.

Supplementary Figure 17

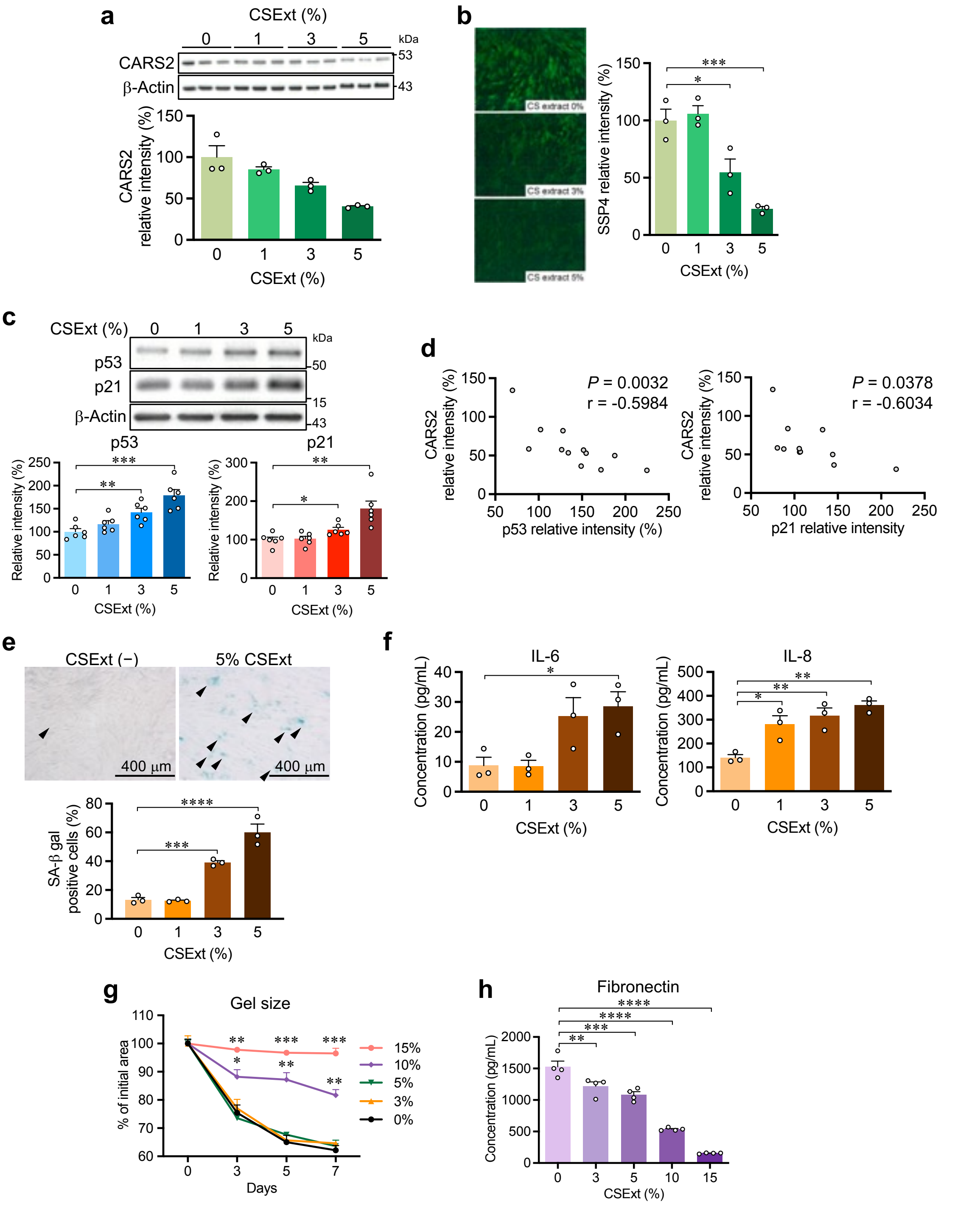

Supplementary Fig. 17 Increased levels of senescence markers caused by reduction of supersulphides in human lung fibroblasts after CSExt exposure.

a, Human foetal lung fibroblasts-1 (HFL-1) cells were exposed to various concentrations of CSExt for 10 days and whole cell lysates were harvested. CARS2 protein levels in cells

were measured by using Western blotting, with β-actin used as the loading control. Each dot represents data from an individual cells (n = 3 per group). **b**, Amounts of supersulphides in CSExt-exposed cells were analyzed by measuring the fluorescence intensity of SSP4 (left panel). Quantification of the fluorescence intensity of SSP4 in cells (right panel) (n = 3). P = 0.022 and 0.0009. **c**, p53 and p21 protein levels in CSExt-exposed cells were evaluated by using Western blotting. Representative Western blot of p53 and p21 appears in the upper panel. Quantification of p53 and p21 protein levels in cells (n = 6) appears in the lower panels. P = 0.0035 (p53), 0.0002 (p53), = 0.019 (p21), and 0.0031 (p21). **d**, Correlations between protein levels of CARS2 and p53 (left panel) or CARS2 and p21 (right panel) in CSExt-exposed cells were evaluated by means of Spearman's rank test. **e**, HFL-1 cells treated with 5% CSExt were grown in 6-well culture plates for 10 days, followed by senescence-associated β-galactosidase (SA-β-gal) staining. Upper panels provide representative images of SA-β-gal-positive cells (arrowheads). Lower panel provides quantification of SA-β-gal-positive cells. P = 0.0008 and P < 0.0001. Scale bars, 400 μm. **f**, Concentrations of senescence-associated secretory phenotype components, IL-6 (left panel) and IL-8 (right panel), in supernatants were quantified by means of the enzyme-linked immunosorbent assay (ELISA) (n = 3). P = 0.047 (IL-6), 0.025 (IL-8, 1%), 0.007 (IL-8, 3%), and 0.0017 (IL-8, 5%). **g**, Fibroblast-mediated tissue repair function was evaluated by using a 3D collagen gel contraction assay. Collagen gels were treated with various concentrations of CSExt, and gel size was evaluated on day 5 (n = 3, each group). P = 0.0015 (15%, 3 days), 0.0003 (15%, 5 days), 0.0002 (15%, 7 days), 0.028 (10% 3 days), 0.0029 (10%, 5 days), 0.0017 (10%, 7 days). **h**, Fibronectin concentration in floating medium was quantified via ELISA (n = 3). P = 0.0096 (3%), 0.0004 (5%), < 0.0001 (10%), and < 0.0001 (15%). Data are means ± s.d. $^{*}P < 0.05$, $^{**}P < 0.01$, $^{***}P < 0.001$, $^{****}P < 0.0001$. Source data are provided as a Source Data file.

Supplementary Figure 18

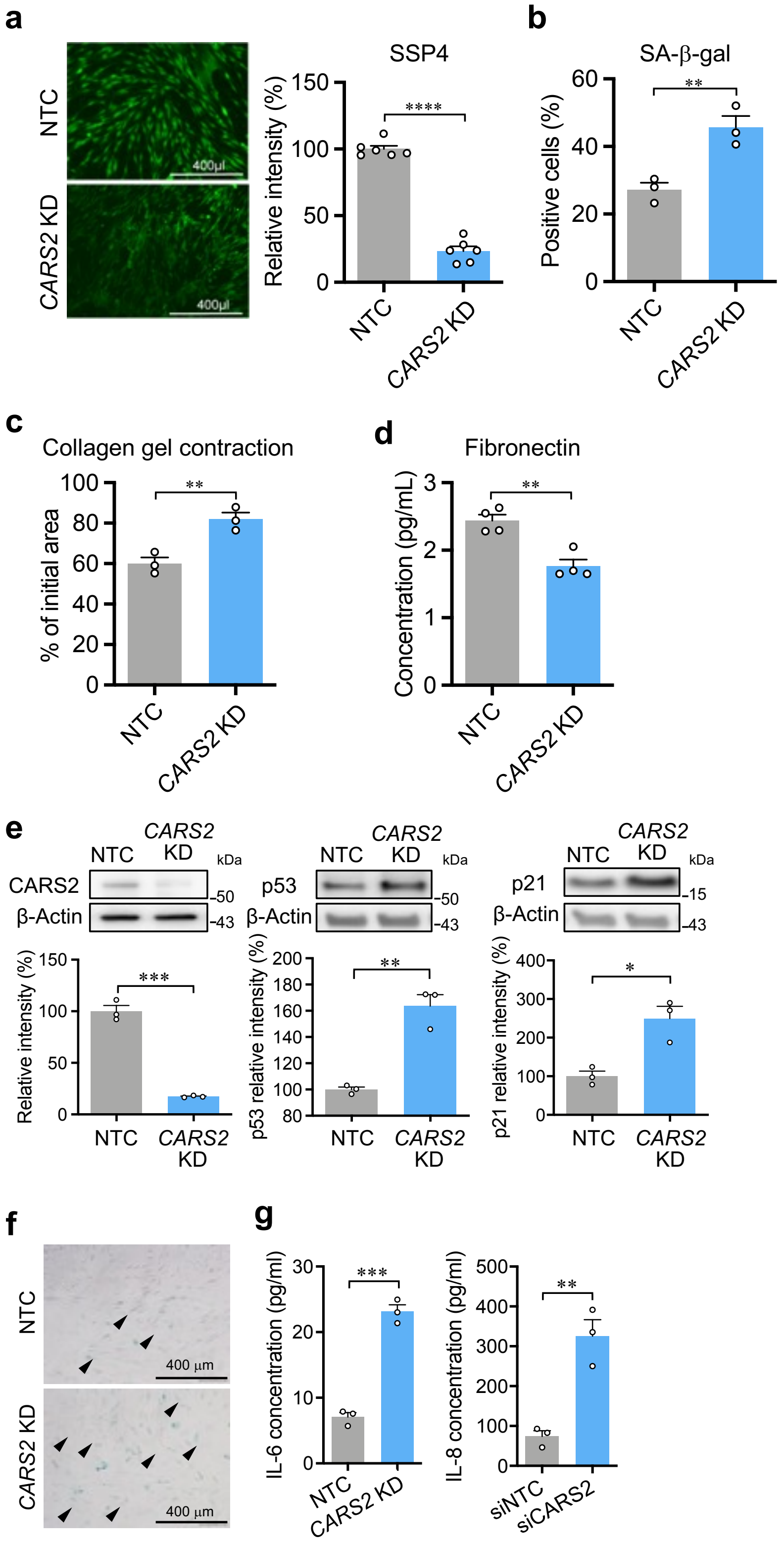

Supplementary Fig. 18 Increased levels of senescence markers caused by reduction of

supersulphides in human lung fibroblasts after *CARS2* knockdown.

a, HFL-1 cells were transfected with *CARS2* siRNA, and amounts of intracellular supersulphides were determined by measuring SSP4 fluorescence intensity (n = 6). Representative fluorescence images of SSP4 are shown. NTC, non-targeting control. P < 0.0001. Scale bars, 400 μm. **b**, HFL-1 cells transfected with *CARS2* siRNA were exposed to 3% CSExt for 7 days. Numbers of SA-β-gal-positive cells were counted (n = 3). P = 0.0097. **c**, Collagen gel contraction assay of HFL-1 cells treated with *CARS2* siRNA, followed by 10% CSExt exposure. Gel sizes were measured on day 5 (n = 3). P = 0.0083. **d**, Fibronectin concentration in the floating medium was quantified with ELISA. P = 0.0022. **e**, CARS2 (left panel), p53 (middle panel), and p21 (right panel) protein levels in HFL-1 cells transfected with *CARS2* siRNA were confirmed by using Western blotting; quantitative results appear in lower panels (n = 3). P = 0.0001, 0.0021, 0.012. **f**, Representative images of SA-β-gal-positive staining in CSExt-exposed siRNA-transfected cells. Arrowheads indicate SA-β-gal-positive cells. Scale bars, 400 μm. **g**, Concentrations of IL-6 (left panel) and IL-8 (right panel) in supernatants quantified by using ELISA (n = 3). P = 0.0002 and 0.0044. Data are means ± s.d. $^{*}P < 0.05$, $^{**}P < 0.01$, $^{***}P < 0.001$, $^{****}P < 0.0001$. Source data are provided as a Source Data file.

Supplementary Table 1 MRM parameters of target peptides for SARS-CoV-2 detection used for LC-ESI-MS/MS analyses.

Virus component	Peptide	Precursor ion (*m/z*)	Product ion (*m/z*)	Collision energy (V)
Nucleoprotein	DGIIWVATEGALNTPK	842.9489	1001.5262	-28
	DGIIWVATEGALNTP**K***	846.956	1009.5404	-28
	AYNVTQAFGR	563.7856	778.4206	-19
Spike protein	IADYNYK	443.7189	773.3464	-16
	HTPINLVR	475.2825	711.4512	-20

*Isotope labeled

Supplementary References

1. Ida, T. et al. Reactive cysteine persulfides and S-polythiolation regulate oxidative stress and redox signaling. *Proc. Natl. Acad. Sci. USA* **111**, 7606-7611 (2014).
2. Ono, K. et al. Redox chemistry and chemical biology of H_2S, hydropersulfides, and derived species: implications of their possible biological activity and utility. *Free Radic. Biol. Med.* **77**, 82-94 (2014).
3. Saund, S. S. et al. The chemical biology of hydropersulfides (RSSH): chemical stability, reactivity and redox roles. *Arch. Biochem. Biophys.* **588**, 15-24 (2015).
4. Fukuto, J. M. et al. Biological hydropersulfides and related polysulfides – a new concept and perspective in redox biology. *FEBS Lett.* **592**, 2140-2152 (2018).
5. Zhang, T. et al. Enhanced cellular polysulfides negatively regulate TLR4 signaling and mitigate lethal endotoxin shock. *Cell Chem. Biol.* **26**, 686-698.e4 (2019).
6. Zivanovic, J. et al. Selective persulfide detection reveals evolutionarily conserved antiaging effects of S-Sulfhydration. *Cell Metab.* **30**, 1152-1170 (2019).
7. Cuevasanta, E. et al. Reaction of hydrogen sulfide with disulfide and sulfenic acid to form the strongly nucleophilic persulfide. *J. Biol. Chem.* **290**, 26866-26880 (2015).
8. Li, H. et al. Using resonance synchronous spectroscopy to characterize the reactivity and electrophilicity of biologically relevant sulfane sulfur. *Redox Biol.* **24**, 101179 (2019).
9. Everett, S. A. & Wardman, P. Perthiols as antioxidants: radical-scavenging and prooxidative mechanisms. *Methods Enzymol.* **251**, 55-69 (1995).
10. Fukuto, J. M. et al. Small molecule signaling agents: the integrated chemistry and biochemistry of nitrogen oxides, oxides of carbon, dioxygen, hydrogen sulfide, and their derived species. *Chem. Res. Toxicol.* **25**, 769-793 (2012).
11. Chauvin, J.-P. R., Griesser, M. & Pratt, D. A. Hydropersulfides: h-atom transfer agents par excellence. *J. Am. Chem. Soc.* **139**, 6484-6493 (2017).
12. Sawa, T. et al. Protein S-guanylation by the biological signal 8-nitroguanosine 3′,5′-cyclic monophosphate. *Nat. Chem. Biol.* **3**, 727-735 (2007).
13. Ihara, H. et al. Exposure to electrophiles impairs reactive persulfide-dependent redox signaling in neuronal cells. *Chem. Res. Toxicol.* **30**, 1673-1684 (2017).
14. Hamid, H. A. et al. Polysulfide stabilization by tyrosine and hydroxyphenyl-containing derivatives that is important for a reactive sulfur metabolomics analysis. *Redox Biol.* **21**, 101096 (2019).

15. Kasamatsu, S. et al. High-precision sulfur metabolomics innovated by a new specific probe for trapping reactive sulfur species. *Antioxid. Redox Signal.* **34**, 1407-1419 (2021).
