# Supplementary material for: Supersulphides provide airway protection in viral and chronic lung diseases
Source: Nat Commun. 2023 Jul 25;14:4476. doi: 10.1038/s41467-023-40182-4 (PMC10368687; doi:10.1038/s41467-023-40182-4)
Supplement: Supplementary file 3 — Reporting summary [file 41467_2023_40182_MOESM3_ESM.pdf]

nature portfolio

Corresponding author(s): TAKAAKI AKAIKE

Last updated by author(s): Jun 11, 2023

Reporting Summary

Nature Portfolio wishes to improve the reproducibility of the work that we publish. This form provides structure for consistency and transparency in reporting. For further information on Nature Portfolio policies, see our Editorial Policies and the Editorial Policy Checklist.

Statistics

For all statistical analyses, confirm that the following items are present in the figure legend, table legend, main text, or Methods section.

n/a	Confirmed	
☐	☒	The exact sample size (*n*) for each experimental group/condition, given as a discrete number and unit of measurement
☐	☒	A statement on whether measurements were taken from distinct samples or whether the same sample was measured repeatedly
☐	☒	The statistical test(s) used AND whether they are one- or two-sided<br>*Only common tests should be described solely by name; describe more complex techniques in the Methods section.*
☒	☐	A description of all covariates tested
☐	☒	A description of any assumptions or corrections, such as tests of normality and adjustment for multiple comparisons
☐	☒	A full description of the statistical parameters including central tendency (e.g. means) or other basic estimates (e.g. regression coefficient) AND variation (e.g. standard deviation) or associated estimates of uncertainty (e.g. confidence intervals)
☐	☒	For null hypothesis testing, the test statistic (e.g. *F*, *t*, *r*) with confidence intervals, effect sizes, degrees of freedom and *P* value noted<br>*Give P values as exact values whenever suitable.*
☒	☐	For Bayesian analysis, information on the choice of priors and Markov chain Monte Carlo settings
☒	☐	For hierarchical and complex designs, identification of the appropriate level for tests and full reporting of outcomes
☐	☒	Estimates of effect sizes (e.g. Cohen's *d*, Pearson's *r*), indicating how they were calculated

Our web collection on statistics for biologists contains articles on many of the points above.

Software and code

Policy information about availability of computer code

Data collection	luminescent image analyser (LAS-4000; Fujifilm), CT scanner (LaTheta LCT-200; Hitachi).
Data analysis	Statistical analysis was performed using GraphPad Prism 9 (GraphPad Software, La Jolla, CA), ImageJ 1.52v software (National Institutes of Health).

For manuscripts utilizing custom algorithms or software that are central to the research but not yet described in published literature, software must be made available to editors and reviewers. We strongly encourage code deposition in a community repository (e.g. GitHub). See the Nature Portfolio guidelines for submitting code & software for further information.

Data

Policy information about availability of data

All manuscripts must include a data availability statement. This statement should provide the following information, where applicable:

- Accession codes, unique identifiers, or web links for publicly available datasets
- A description of any restrictions on data availability
- For clinical datasets or third party data, please ensure that the statement adheres to our policy

All data generated and analyzed in this study are included in this article and its Supplementary Information files. Source data are provided with this paper.

Research involving human participants, their data, or biological material

Policy information about studies with human participants or human data. See also policy information about sex, gender (identity/presentation), and sexual orientation and race, ethnicity and racism.

Reporting on sex and gender	In this study, all subjects' sex were consistent with their self-reported gender. For primary human lung-resident cell study, all 15 subjects were male in the group of the patients with COPD; in the group of 15 never-smoker patients without COPD, 4 subjects were male and 11 subjects were female; in the group of 12 ex-smoker patients without COPD, 7 subjects were male and 5 subjects were female. Because to investigate the differences of the expression of CARS2 and the mitochondrial membrane potential between the primary airway or lung cells derived from the patients with or without COPD, we did not perform the sex- and gender-based analysis. To obtain EBC, 22 subjects for healthy volunteer, 22 subjects for COVID-19. Sex or gender analysis was not performed.
Reporting on race, ethnicity, or other socially relevant groupings	All participants are Japanese.
Population characteristics	For primary human lung-resident cell study, the ages of 15 patients with COPD ranged from 51 to 78 (median: 68); were male; the ages of 15 never-smoker patients without COPD ranged from 49 to 80 (median: 65); the ages of 12 ex-smoker patients without COPD ranged from 47 to 80 (median: 63). All 22 subjects for healthy volunteer and 22 subjects for COVID-19 were over 18.
Recruitment	For primary human lung-resident cell study, the lung cancer patients who underwent pneumonectomy and wish to participate voluntary in our study were recruited between January 2013 and July 2019. The patients with COPD satisfied the diagnostic criteria of the Global Initiative for Chronic Obstructive Lung Disease (GOLD) report. Current smokers were excluded. All ex-smokers had quit smoking for at least 1 year before the surgery. 15 patients with COPD participated voluntarily and randomly in primary human lung-resident cell study. We then examined the primary cells derived from age-matched 15 never-smoker patients without COPD and 12 ex-smoker patients without COPD because we tried to reduce the effect of the covariate of age. To obtain EBC, 22 subjects for healthy volunteer and 22 subjects for COVID-19 participated voluntarily and randomly.
Ethics oversight	Written informed consent was obtained from all subjects who participated. All experiments were approved by the Ethics Committee of the Institutional Review Board of Tohoku University Graduate School of Medicine (2022-1-254-1).

Note that full information on the approval of the study protocol must also be provided in the manuscript.

Field-specific reporting

Please select the one below that is the best fit for your research. If you are not sure, read the appropriate sections before making your selection.

☒ Life sciences ☐ Behavioural & social sciences ☐ Ecological, evolutionary & environmental sciences

For a reference copy of the document with all sections, see nature.com/documents/nr-reporting-summary-flat.pdf

Life sciences study design

All studies must disclose on these points even when the disclosure is negative.

Sample size	No sample size calculation was performed. Sample sizes (3-5 samples for cell line analyses, 8-12 samples for mouse experiment, 20-40 samples for human samples) were chosen based on previous experience and on what is common practice in the field. A smaller sample size of 3 was used in a limited number of experiments when the availability of sample is scarce.
Data exclusions	No data was excluded.
Replication	All experiments were independently repeated as described in the legends and methods and were reliably reproduced.
Randomization	We used randomized paired (a.k.a. matched pairs) design. We paired animals to two or more treatment groups on the basis of similar weight, age, delivery date, and when possible holding cage. For other experiments, randomization was not a relevant feature as we applying a uniform set of techniques.
Blinding	Histological evaluation of human tumor samples were blindly performed. For other experiments, blinding was not conducted during experiments because each experiment was performed by a single investigator and because collected data are quantitative and not influenced by investigator's bias.

Behavioural & social sciences study design

All studies must disclose on these points even when the disclosure is negative.

Study description	*Briefly describe the study type including whether data are quantitative, qualitative, or mixed-methods (e.g. qualitative cross-sectional, quantitative experimental, mixed-methods case study).*
Research sample	*State the research sample (e.g. Harvard university undergraduates, villagers in rural India) and provide relevant demographic information (e.g. age, sex) and indicate whether the sample is representative. Provide a rationale for the study sample chosen. For studies involving existing datasets, please describe the dataset and source.*
Sampling strategy	*Describe the sampling procedure (e.g. random, snowball, stratified, convenience). Describe the statistical methods that were used to predetermine sample size OR if no sample-size calculation was performed, describe how sample sizes were chosen and provide a rationale for why these sample sizes are sufficient. For qualitative data, please indicate whether data saturation was considered, and what criteria were used to decide that no further sampling was needed.*
Data collection	*Provide details about the data collection procedure, including the instruments or devices used to record the data (e.g. pen and paper, computer, eye tracker, video or audio equipment) whether anyone was present besides the participant(s) and the researcher, and whether the researcher was blind to experimental condition and/or the study hypothesis during data collection.*
Timing	*Indicate the start and stop dates of data collection. If there is a gap between collection periods, state the dates for each sample cohort.*
Data exclusions	*If no data were excluded from the analyses, state so OR if data were excluded, provide the exact number of exclusions and the rationale behind them, indicating whether exclusion criteria were pre-established.*
Non-participation	*State how many participants dropped out/declined participation and the reason(s) given OR provide response rate OR state that no participants dropped out/declined participation.*
Randomization	*If participants were not allocated into experimental groups, state so OR describe how participants were allocated to groups, and if allocation was not random, describe how covariates were controlled.*

Ecological, evolutionary & environmental sciences study design

All studies must disclose on these points even when the disclosure is negative.

Study description	*Briefly describe the study. For quantitative data include treatment factors and interactions, design structure (e.g. factorial, nested, hierarchical), nature and number of experimental units and replicates.*
Research sample	*Describe the research sample (e.g. a group of tagged Passer domesticus, all Stenocereus thurberi within Organ Pipe Cactus National Monument), and provide a rationale for the sample choice. When relevant, describe the organism taxa, source, sex, age range and any manipulations. State what population the sample is meant to represent when applicable. For studies involving existing datasets, describe the data and its source.*
Sampling strategy	*Note the sampling procedure. Describe the statistical methods that were used to predetermine sample size OR if no sample-size calculation was performed, describe how sample sizes were chosen and provide a rationale for why these sample sizes are sufficient.*
Data collection	*Describe the data collection procedure, including who recorded the data and how.*
Timing and spatial scale	*Indicate the start and stop dates of data collection, noting the frequency and periodicity of sampling and providing a rationale for these choices. If there is a gap between collection periods, state the dates for each sample cohort. Specify the spatial scale from which the data are taken*
Data exclusions	*If no data were excluded from the analyses, state so OR if data were excluded, describe the exclusions and the rationale behind them, indicating whether exclusion criteria were pre-established.*
Reproducibility	*Describe the measures taken to verify the reproducibility of experimental findings. For each experiment, note whether any attempts to repeat the experiment failed OR state that all attempts to repeat the experiment were successful.*
Randomization	*Describe how samples/organisms/participants were allocated into groups. If allocation was not random, describe how covariates were controlled. If this is not relevant to your study, explain why.*
Blinding	*Describe the extent of blinding used during data acquisition and analysis. If blinding was not possible, describe why OR explain why blinding was not relevant to your study.*

Did the study involve field work? ☐ Yes ☐ No

Field work, collection and transport

Field conditions	*Describe the study conditions for field work, providing relevant parameters (e.g. temperature, rainfall).*
Location	*State the location of the sampling or experiment, providing relevant parameters (e.g. latitude and longitude, elevation, water depth).*
Access & import/export	*Describe the efforts you have made to access habitats and to collect and import/export your samples in a responsible manner and in compliance with local, national and international laws, noting any permits that were obtained (give the name of the issuing authority, the date of issue, and any identifying information).*
Disturbance	*Describe any disturbance caused by the study and how it was minimized.*

Reporting for specific materials, systems and methods

We require information from authors about some types of materials, experimental systems and methods used in many studies. Here, indicate whether each material, system or method listed is relevant to your study. If you are not sure if a list item applies to your research, read the appropriate section before selecting a response.

Materials & experimental systems

n/a	Involved in the study	
☐	☒	Antibodies
☐	☒	Eukaryotic cell lines
☐	☐	Palaeontology and archaeology
☐	☒	Animals and other organisms
☒	☐	Clinical data
☒	☐	Dual use research of concern
☒	☐	Plants

Methods

n/a	Involved in the study	
☒	☐	ChIP-seq
☒	☐	Flow cytometry
☒	☐	MRI-based neuroimaging

Antibodies

Antibodies used	rabbit polyclonal anti-CARS1 antibody (1:5,000 dilution, HPA002383; Sigma-Aldrich), rabbit polyclonal anti-human CARS2 antibody (1:2,000 dilution, HPA043935; Sigma-Aldrich), anti-mouse CARS2 antibody (1:5,000 dilution), mouse monoclonal anti-MTCO1 antibody (1:5,000 dilution, ab14705; Abcam), mouse monoclonal anti-SDHA antibody (1:5,000 dilution, ab14715; Abcam), mouse monoclonal anti-p53 antibody (1:200 dilution, 2Q366 ; Santa Cruz Biotechnology), rabbit monoclonal anti-p21 antibody (1:2,000 dilution, 2947 ; Cell Signaling Technology), mouse monoclonal anti-beta-actin antibody (1:10,000 dilution, sc-1615; Santa Cruz Biotechnology), and rabbit polyclonal anti-3-NT antibody (1:1,000 dilution, 06-284 ; Upstate Biotechnology), and rabbit polyclonal anti-GAPDH antibody (1:5000 dilution, sc5778, Santa Cruz Biotechnology), horseradish peroxidase-linked antibody directed against rabbit IgG (1:10,000, Cell Signaling, 7074), mouse monoclonal anti-beta-actin antibody (1:10,000 dilution, sc-1615; Santa Cruz Biotechnology), rabbit anti-cleaved caspase-3 antibody (1:600 dilution;#9661, Cell Signaling Technology), The specific rat polyclonal antibody for mouse CARS2 was produced by immunizing rats with recombinant mouse CARS2 and by immunoaffinity purification. This CARS2 was used in previous our paper (https://doi.org/10.1038/s41467-017-01311-y).
Validation	All antibodies except mouse CARS2 antibody are commercially available. Antibodies employed here in our manuscript were previously reported and routinely used for the application used.

Eukaryotic cell lines

Policy information about cell lines and Sex and Gender in Research

Cell line source(s)	MDCK cells (ATCC, CCL-34), DBT cells(ATCC, CRL-2020), and Human fetal lung fibroblasts (HFL-1) (CCL-153) VeroE6/TMPRSS2 cell lines (No. JCRB1819). Human embryonic kidney cell HEK293T cells (ATCC, CRL-3216).
Authentication	None of the cell lines used were authenticated.
Mycoplasma contamination	All cells were tested negative for mycoplasma.
Commonly misidentified lines (See ICLAC register)	No commonly misidentified cell lines were used in this study.

Palaeontology and Archaeology

Specimen provenance	*Provide provenance information for specimens and describe permits that were obtained for the work (including the name of the*

Specimen provenance	*issuing authority, the date of issue, and any identifying information). Permits should encompass collection and, where applicable, export.*
Specimen deposition	*Indicate where the specimens have been deposited to permit free access by other researchers.*
Dating methods	*If new dates are provided, describe how they were obtained (e.g. collection, storage, sample pretreatment and measurement), where they were obtained (i.e. lab name), the calibration program and the protocol for quality assurance OR state that no new dates are provided.*

☐ Tick this box to confirm that the raw and calibrated dates are available in the paper or in Supplementary Information.

Ethics oversight	*Identify the organization(s) that approved or provided guidance on the study protocol, OR state that no ethical approval or guidance was required and explain why not.*

Note that full information on the approval of the study protocol must also be provided in the manuscript.

Animals and other research organisms

Policy information about studies involving animals; ARRIVE guidelines recommended for reporting animal research, and Sex and Gender in Research

Laboratory animals	C57BL6J mice and Syrian hamsters were purchased from Japan SLC, Inc. Seven- to 17-week-old mice and Seven-week-old Syrian hamsters were used for experiments.
Wild animals	The study did not involve wild animals.
Reporting on sex	Syrian hamster: Male, C57BL6J mice: Male and Female.
Field-collected samples	The study did not involve the samples collected in the field.
Ethics oversight	All experimental procedures conformed to the Regulations for Animal Experiments and Related Activities at Tohoku University, were reviewed by the Institutional Laboratory Animal Care and Use Committee of Tohoku University, and were finally approved by the President of Tohoku University. Mice and hamsters were housed in a specific pathogen-free facility and maintained under constant temperature (24 ºC), humidity (40%), and light cycle, with food and water provided ad libitum.

Note that full information on the approval of the study protocol must also be provided in the manuscript.

Dual use research of concern

Policy information about dual use research of concern

Hazards

Could the accidental, deliberate or reckless misuse of agents or technologies generated in the work, or the application of information presented in the manuscript, pose a threat to:

No	Yes	
☒	☐	Public health
☒	☐	National security
☒	☐	Crops and/or livestock
☒	☐	Ecosystems
☒	☐	Any other significant area

Experiments of concern

Does the work involve any of these experiments of concern:

No	Yes	
☒	☐	Demonstrate how to render a vaccine ineffective
☒	☐	Confer resistance to therapeutically useful antibiotics or antiviral agents
☒	☐	Enhance the virulence of a pathogen or render a nonpathogen virulent
☒	☐	Increase transmissibility of a pathogen
☒	☐	Alter the host range of a pathogen
☒	☐	Enable evasion of diagnostic/detection modalities
☒	☐	Enable the weaponization of a biological agent or toxin
☒	☐	Any other potentially harmful combination of experiments and agents
